# The Role of Soil Microbial Diversity in the Conservation of Native Seed Bacterial Microbiomes

**DOI:** 10.3390/microorganisms10040750

**Published:** 2022-03-30

**Authors:** Ankush Chandel, Ross Mann, Jatinder Kaur, Sally Norton, Desmond Auer, Jacqueline Edwards, German Spangenberg, Timothy Sawbridge

**Affiliations:** 1 Agriculture Victoria, AgriBio Centre for AgriBioscience, Bundoora, VIC 3083, Australia; ross.mann@agriculture.vic.gov.au (R.M.); jatinder.kaur@agriculture.vic.gov.au (J.K.); desmond.auer@agriculture.vic.gov.au (D.A.); jacky.edwards@agriculture.vic.gov.au (J.E.); germancspangenberg@gmail.com (G.S.); tim.sawbridge@agriculture.vic.gov.au (T.S.); 2 School of Applied Systems Biology, La Trobe University, Bundoora, VIC 3083, Australia; 3 Agriculture Victoria, Australian Grains Genebank, Horsham, VIC 3400, Australia; sally.norton@agriculture.vic.gov.au

**Keywords:** 16S rRNA, seedbank, seed vault, *Glycine clandestina*, seed microbiome, bacterial diversity, epiphytes, endophytes, soil microbial diversity

## Abstract

Research into understanding the structure, composition and vertical transmission of crop seed microbiomes has intensified, although there is much less research into the seed microbiomes of crop wild relatives. Our previous study showed that the standard seed storage procedures (e.g., seed drying and storage temperature) can influence the seed microbiome of domesticated *Glycine max*. In this study, we characterized the seed microbiota of *Glycine clandestina*, a perennial wild relative of soybean (*G. max* (L.) Merr.) to expand our understanding about the effect of other storage procedures such as the periodic regeneration of seed stocks to bulk up seed numbers and secure viability on the seed microbiome of said seed. The *G. clandestina* microbiota was analysed from Generation 1 (G1) and Generation 2 (G2) seed and from mature plant organs grown in two different soil treatments T (treatment [native soil + potting mix]) and C (control [potting mix only]). Our dataset showed that soil microbiota had a strong influence on next generation seed microbiota, with an increased contribution of root microbiota by 90% and seed transmissibility by 36.3% in G2 (T) seed. Interestingly, the G2 seed microbiota primarily consisted of an initially low abundance of taxa present in G1 seed. Overall, our results indicate that seed regeneration can affect the seed microbiome composition and using native soil from the location of the source plant can enhance the conservation of the native seed microbiota.

## 1. Introduction

The plant microbiome consists of a multitude of microbes that have important functions in enhancing the health and productivity of the host plant in their natural environments [1]. Plant organs are colonized by different microbial communities, either categorised as epiphytes that remain on the surface of plant organs, or endophytes which inhabit and live inside plant tissues [2,3]. Many plant-associated microbes are recruited via horizontal transfer from local microbial habitats such as soil, as well as the external environments of leaves, flowers, fruit and seed [2]. A subset of microbes are also vertically transmitted through vegetative propagation and sexual reproduction via seed [4].

Plant seed provides a stable environment for a microbial community protected by the seed coat [5] and enables the vertical transmission of members of the seed microbial community to inhabit the next generation [6,7]. The potential benefits of seed-borne microbes, such as enhanced seed germination under different biotic-abiotic stress conditions, has been suitably demonstrated elsewhere [8,9]. Moreover, these microbes can benefit the host plant by promoting plant growth and providing biocontrol of pathogenic activity [10,11]. Seed microbes can also drive the assembly of root microbiota due to their enhanced ability to colonize the rhizosphere [12,13]. For instance, Moroenyane et al. [14] demonstrated that the microbiome composition of soybean plant compartments (root and shoot) was primarily modulated by the seed microbial communities, not by the soil microbial communities, even though the seed microbiome was initially disrupted using X-ray irradiation before a 14-day incubation in sterile sand. In contrast, Rochefort et al. [4] showed that the early seedling root microbiota composition was influenced more by the soil than the seed microbiota. They also observed that the transmission rate of rare and intermediate seed-borne and soil-borne taxa into seedlings was higher than highly-abundant taxa [4]. With such contradictions present in the literature, knowledge of the modes of transmission of microbes to the next generation is essential to implement effective strategies for plant microbiome engineering through modifications of native seed or native soil microbiota in sustainable agriculture [4,15]. 

Plant domestication and intense agricultural practices have resulted in variations in the composition of the inherent microbiome from wild crop relatives, usually with a loss of microbial diversity in the domesticated crop [16,17]. This has been highlighted in recent studies which indicated significant changes in the seed microbiota of cultivated crops, with wild progenitors shown to harbor different microbial communities compared to that of domesticated crops [5,18,19]. For instance, different bacterial communities were associated with the rhizosphere of wild and domesticated common bean, and, notably, these changes were linked to differences in the root length [17]. To date, most studies have focused on the use of the genetic diversity of native plants for developing more resilient domestic crop cultivars [20]. However, the efficacy of native seed microbes in enhancing modern crops’ health and productivity has rarely been explored [21]. The conservation of native seed microbes has the potential to identify key components of this untapped indigenous microbial diversity and their possible metabolic benefits in enhancing domesticated crop plant productivity, with possibly both environmental and human health benefits [22].

Seed Vaults conserve the seed germplasm of numerous crop cultivars and their closest wild relatives for future crop improvement and essential scientific research work following International Genebank guidelines by acting as a secondary backup for every seedbank in the world [23]. There is little literature on the effects of practices used by seed banks to maintain their stocks of seed or on the microbiomes of the stored seed.

In this study, we describe the seed microbiome of *Glycine clandestina*, a perennial wild crop relative of the domesticated soybean, *Glycine max* (L. Merr) [20]. We studied the effect of seed regeneration on the composition and diversity of the *G. clandestina* seed microbiome using the standard bulk up method followed by the Australian Grains Genebank, and a modification of this method in which the standard potting mix was supplemented with native soil from where the seed was harvested. While recognizing the complexity of the soil microbiome [24,25], the focus of this study was to examine using a native soil inoculum to enhance vertical transmission of seed bacterial communities to progeny seed under greenhouse conditions as a method that could be used by seedbanks to preserve wild seed microbiomes, and this study focuses on examining the plant bacterial microbiomes. The spatial dynamics of the bacterial communities associated with plant tissues (root, shoot, leaf) during the seed maturation stage were examined to compare to the bacteria contained in the next generation seed to assess possible transmission routes for intergenerational bacterial transmission. Therefore, in the present study, we have used native soil inoculum to provide an experimental basis for our understanding of the implication of this method by seed vaults to conserve the microbial communities associated with native plant seeds. 

## 2. Materials and Methods

### 2.1. Glycine Seed Collection

*Glycine clandestina* plants were identified in the Dandenong Ranges National Park (−37.8809083, 145.3163306) guided by the online database “Atlas of Living Australia”. https://bie.ala.org.au/search?q=Glycine+clandestina (accessed on 22 January 2020). Seed pods were collected under a permit approved by Parks Victoria, placed into a paper bag, and allowed to dry and shatter naturally under room temperature conditions. Seeds were then separated and stored in a clean paper bag at room temperature in the laboratory.

### 2.2. Plant Growth and Sample Collection

**Figure 1:**
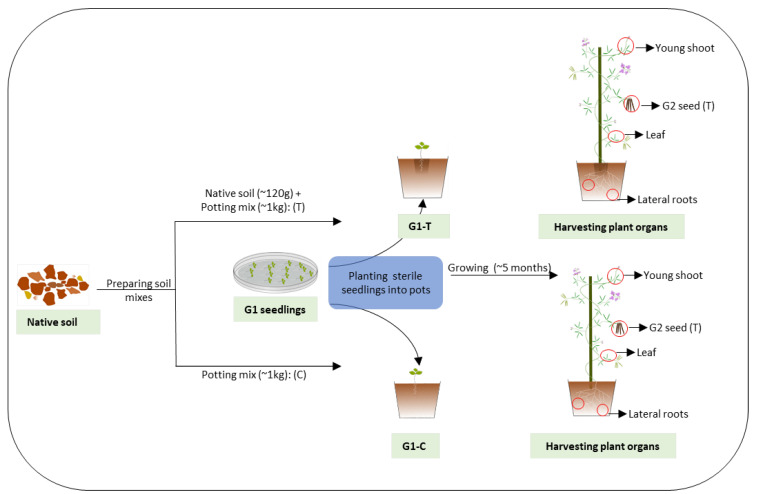
Schematic representation of the greenhouse experimental set up.

*G. clandestina* seeds were germinated as per Chandel et al. [26] until they reached the trifoliate stage (~12 days) (Supplementary Figure S1). A subset was harvested for DNA extraction and the remainder planted into pots (200 mm × 190 mm) in a greenhouse. For the greenhouse setup, fresh topsoil samples associated with the source plant were collected under the same permit to a depth of 10–15 cm at the same locations from the Dandenong Ranges and stored in a plastic bag. The soil was transported immediately to the AgriBio Research Centre greenhouse on ice. After removal of roots and debris, the soil was homogenized by hand mixing, then about 120 g of soil was allocated to a pit created in the above pots containing about one kg of standard native potting mix (Scott’s Osmocote Native Premium Potting Mix). The pot treatments were then designated as T (treatment [soil + potting mix], G1-T) and C (control [potting mix only], G1-C). The seedlings (Generation 1, G1) were then transplanted into either of the soil mixes in triplicate and grown with a 14-h day cycle at 22 °C (growth lights were on when outside light intensity dropped below 170 W/m^2^) followed by a 10-h night cycle (growth lights turned off) at 14 °C in the greenhouse for about five months (Figure 1). Generation 2 (G2) seeds were harvested over a two week period from three plant replicates for each soil treatment. The plant organs [(root, shoot, leaf (three to four technical replicates)] from mature plants were harvested by removing single trifoliate leaves of similar size, young shoots (~10 cm without leaf and flower buds), and lateral roots (Figure 1). Roots were washed with an excess amount of sterile phosphate-buffered saline (PBS) and then all plant tissues were kept at −80 °C until processed for DNA extraction. For seedling DNA extraction, the harvested G1 and G2 seedlings were pooled in sets of three seedlings into 1.2 mL QIAGEN collection tubes creating 12 biological replicates for G1 and G2 (T and C) seed, then snap frozen in liquid nitrogen and stored at −80 °C for DNA extraction. 

### 2.3. Microbial DNA Extraction and Amplicon Library Construction

The DNA extraction and amplicon libraries preparation for Illumina sequencing were performed as per Chandel et al. [26]. Paired end sequencing was performed on Miseq v3 (2 × 300 bp v3 chemistry cartridge). All Illumina sequences have been submitted to the NCBI short read Archive (SRA accession PRJNA810761).

### 2.4. Bioinformatic Analysis of 16S rRNA Gene Amplicon Library Sequences

The sequencing data were processed using Qiime2 as per Chandel et al. [26] with the following modifications: Reads were grouped by soil treatment (T and C) and then rarefied to 1680 sequences for microbiome profiling of *G. clandestina* seed [G1 and G2 (T and C) seed] (Supplementary Figure S2) and 1172 sequences for microbiome profiling of *G. clandestina* plant organs [G1 (T and C) plant organs (root, shoot, leaf and G2 seed)]; and G1 seed, [G1 (T and C) plant organs (root, shoot, leaf and G2 seed)] (Supplementary Figure S3). Also, Venn diagrams were plotted in Genedata Expressionist^®^ Analyst™ v.10.0 (Genedata; Basel, Switzerland) by exporting the grouped rarefied feature table at the genus level to determine the number of shared and unique genera across G1 (T and C) plant organs; and G1 seed and G1 (T and C) plant organs.

## 3. Results

### 3.1. 16S rRNA Gene Sequencing

After aligning raw paired-end reads, removing low-frequency features, singletons and plant associated sequences, a total of 1,023,313 sequences were assigned to 536 Amplicon Sequence Variants (ASVs) for microbiome profiling of *G. clandestina* seed [G1 and G2 (T and C) seed] and 371,487 sequences were assigned to 822 ASVs for microbiome profiling of *G. clandestina* plant organs [G1 plant organs and G2 (T and C) seed]. After rarefaction and collapsing biological replicates, the ASV table was assigned to 163 genera for microbiome profiling of *G. clandestina* seed (Supplementary Table S3) and 412 genera for microbiome profiling of *G. clandestina* plant organs (Supplementary Table S4).

### 3.2. Microbiome Profiling. Temporal Variation in the G. clandestina Seed Microbiome

**Figure 2:**
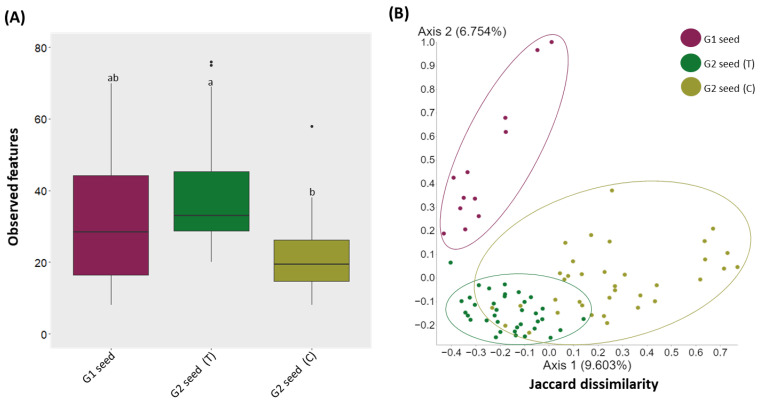
Alpha (observed features) and β-diversity (Jaccard dissimilarity) analyses of the seed microbiota of *G. clandestina* between G1 seed and G2 (T and C) seed. (**A**) “Box-and-Whiskers” plots visualize the observed features for G1 seed and G2 (T and C) seed. Significant differences (*p* ≤ 0.05) were assessed by the Kruskal Wallis pairwise test and are indicated by the lower-case letters. (**B**) PCoA plots showing the distances between the bacterial community composition of G1 and G2 (T and C) seed. Significant differences in bacterial composition were tested using the ANOSIM pairwise test. Different colours of the bars (**A**) and points (**B**) represent the G1 and G2 (T and C) seed represent the plant organs for both soil treatments (T and C).

To assess the effect of soil type on the bacterial diversity and composition of *G. clandestina* seed microbiome, the samples were grouped by treatment, and alpha and beta diversity analyses were performed. Alpha diversity comparison based on observed features showed significant (*H* = 27.4, *p* = 1.10536 × 10^−6^) variations in bacterial diversity. The observed features were significantly (*p* < 0.05) higher in G2 (T) than G2 (C) seed, while no significant differences were observed between G1 seed and G2 (T), nor G1 and G2 (C) seed (Figure 2A and Supplementary Table S5). For β-diversity, the PCoA analysis was conducted based on the Jaccard dissimilarity index in combination with ANOSIM. The bacterial composition was significantly different (*p* = 0.001) between G1 and G2 (T and C) seed, the ANOSIM results explained the higher proportion of the variance between microbiome composition of G1 and G2 (T) seed (*R* = 0.671, *p* = 0.001), G1 and G2 (C) seed (*R* = 0.672, *p* = 0.001). When variance was compared between the G2 (T) and G2 (C) seed, the variation (*R* = 0.339, *p* = 0.001) was lessened, but still significant (Figure 2B and Supplementary Table S6).

### 3.3. G. clandestina Seed Microbiome Composition G1 and G2 (T and C)

**Figure 3:**
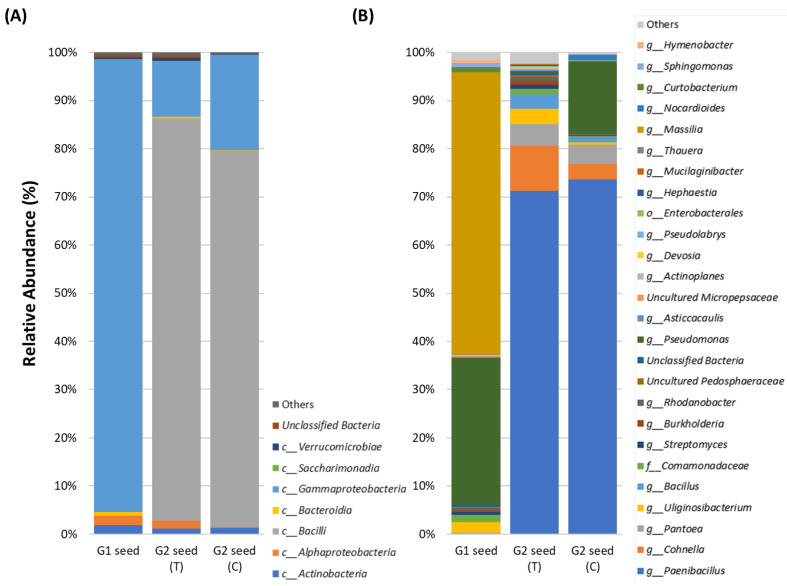
Relative abundance of *G. clandestina* microbiomes across G1, G2 (T) and G2 (C) seed at the class (**A**) and genus level (**B**). Taxa occurring with less than 0.1% relative abundance are shown as “Others”.

The composition of the seed microbiome was influenced by the soil type and generation. The abundance of the most dominant classes was reduced substantially between the G1 seed and G2 (T and C) seed. For instance, in G1 seed, the three most dominant classes were *Gammaproteobacteria* (93.9%), *Actinobacteria* (1.8%) and *Alphaproteobacteria* (1.8%), as opposed to *Bacilli* (83.5%), *Gammaproteobacteria* (11.5%) and *Alphaproteobacteria* (1.61%) in G2 (T), with *Bacilli* (78.0%), *Gammaproteobacteria* (19.8%) and *Actinobacteria* (1.3%) in G2 (C), respectively (Figure 3A and Supplementary Table S1).

Assessing ASVs abundance between seed generations across soil treatments, it was evident that certain ASVs were prominent in G1 seed and G2 (T and C) seed. For instance, in G1 seed, the five most dominant genera were *Massilia* (58.5%), *Pseudomonas* (30.3%), *Uliginosibacterium* (2.1%) and *Comamonadaceae* (1.5%) as opposed to *Paenibacillus* (71.2%), *Cohnella* (9.3%), *Pantoea* (4.4%), *Uliginosibacterium* (3.3%) and *Bacillus* (2.9%) in G2 (T) and *Paenibacillus* (73.6%), *Pseudomonas* (14.8%), *Pantoea* (3.9%), *Cohnella* (3.1%) and *Nocardioides* (1.2%) in G2 (C) seed, respectively (Figure 3B and Supplementary Table S2).

**Figure 4:**
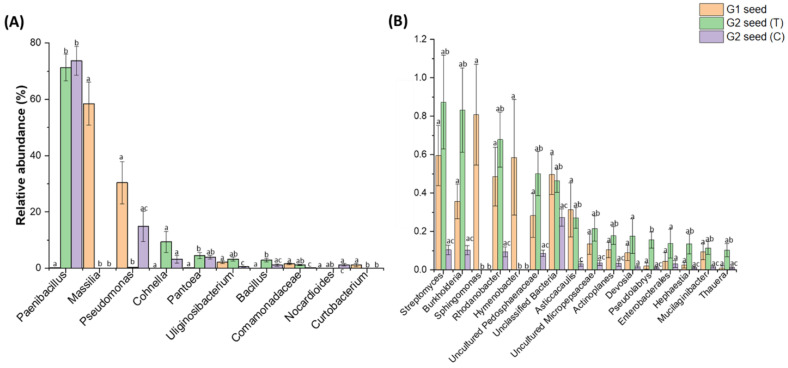
Significant differences (*p* ≤ 0.05) among the bacterial genera with relative abundance (**A**) >1% and (**B**) <1%, >0.1%. between *G. clandestina* seed (G1) and G2 (T and C) seed. The comparisons were determined by using one-way ANOVA followed by Tukey’s test.

The significant differences in bacterial abundance between G2 (T) and G2 (C) seed across two soil treatments were identified using pairwise comparison (Tukey test) of ASVs greater than 1% and less than 1% but greater than 0.1% relative abundance, respectively. In total, there were two (>1%) and 11 ASVs (<1%, >0.1%) that were significantly (*p* < 0.05) higher in G2 (T) seed than in G2 (C) seed, respectively (Figure 4A,B). These ASVs were maintained at levels in the G2 (T) seed similar to that of the G1 seed (Supplementary Table S2). They were *Uliginosibacterium* (Tukey test, *p* = 3.40246 × 10^−4^), *Comamonadaceae* (Tukey test, *p* = 3.7995 × 10^−4^) (Figure 4A), *Streptomyces* (Tukey test, *p* = 0.00398), *Burkholderia* (Tukey test, *p* = 0.00202), *Rhodanobacter* (Tukey test, *p* = 2.80714 × 10^−4^), *Pedosphaeraceae* (Tukey test, *p* = 0.00134), *Asticcacaulis* (Tukey test, *p* = 0.00167), *Micropepsaceae* (Tukey test, *p* = 0.018), *Actinoplanes* (Tukey test, *p* = 0.00701), *Pseudolabrys* (Tukey test, *p* = 0.00162), *Hephaestia* (Tukey test, *p* = 0.03247), *Mucilaginibacter* (Tukey test, *p* = 0.01242) and *Thuera* (Tukey test, *p* = 0.01654) (Figure 4B). Interestingly, the abundance of *Paenibacillus* increased significantly (*p* < 0.05) in G2 (T and C) seed at the expense of most dominant genera *Massilia* (58.5%) which declined significantly (*p* < 0.05) in G2 (T and C) seed (Figure 4A). Additionally, three other genera (>0.1%) including *Sphingomonas*, *Curtobacterium* and *Hymenobacter* were only associated with G1 seeds, while they were not detected in either G2 (T and C) seed (Figure 4B).

### 3.4. Microbiome Profiling, Spatial Variation in the G. clandestina Microbiome

**Figure 5:**
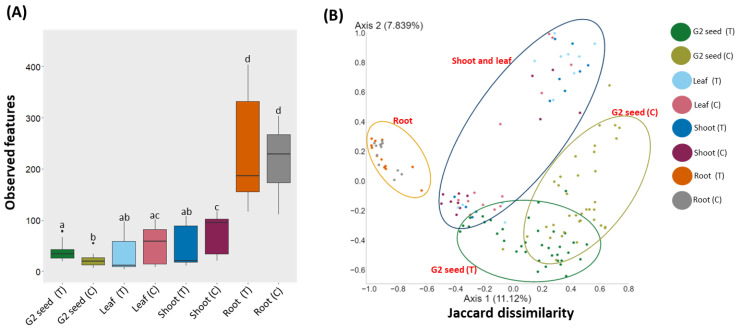
Alpha- (observed features) and β-diversity (Jaccard dissimilarity) analyses of *G. clandestina* plant organs for plants grown in two soil treatments (T and C). (**A**) “Box-and-Whiskers” plots visualize the observed features for plant organs. Significant differences (*p* ≤ 0.05) were assessed by the Kruskal Wallis pairwise test and are indicated by lower-case letters. (**B**) PCoA plots showing the distances between the bacterial community composition of G1 plant organs and G2 seed when grown in two soil treatments (T and C). Significant differences in bacterial composition were tested using the ANOSIM pairwise test. Different colours of the bars (**A**) and points (**B**) represent the G1 plant organs and G2 seed when grown in two soil treatments (T and C).

Alpha diversity, the comparison based on observed features, indicated that bacterial diversity varied significantly (*H* = 85, *p* = 1.30 × 10^−15^) between plant organs. For G1 (T) plants, the observed features were significantly (*p* < 0.05) higher in the root than the above-ground organs (shoot, leaf and G2 (T) seed). No significant differences were observed among the above-ground organs (shoot, leaf, and G2 (T) seed (Figure 5A, Supplementary Table S7). For G1 (C) plants, the observed features were also significantly (*p* < 0.05) higher in the root than in the above-ground organs (shoot, leaf and G2 (C) seed). In contrast to G2 (T), significant differences were observed between the shoot and G2 (C) seed, and the leaf and G2 (C) seed (Figure 5A, Supplementary Table S7). When plant organs are compared between G1 treatment and G1 control plants, the observed features significantly varied between G2 (T) and G2 (C) seed, G1 (T) shoot and G1 (C) shoot. For β-diversity, PCoA analysis identified significant (*p* = 0.001) separation for the bacterial composition of plant organs within and across treatments G2 (T and C). However, no significant separations were observed between leaf and shoot within and across the treatments for G2 (T and C) (Figure 5B, Supplementary Table S8).

### 3.5. G. clandestina Microbiome Composition across G1 Plant Organs and G2 (T and C) Seed

**Figure 6:**
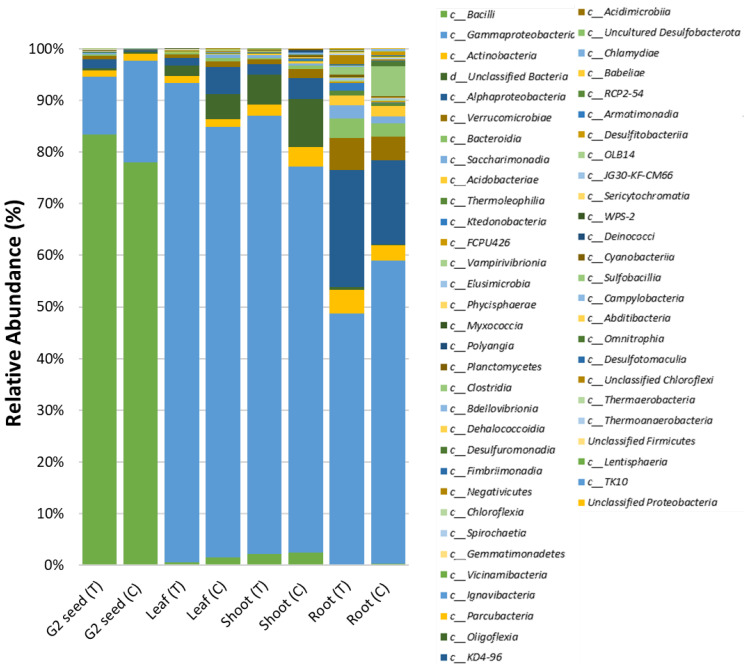
Relative abundance of *G. clandestina* microbiomes across different plant organs (root, shoot, leaf and G2 seed) of mature plants (G1) at class level.

The recruitment and distribution of the original *G. clandestina* microbiome across G1 plant organs (root, shoot, leaf and seed) was found to be influenced by soil type. The most prominent bacterial class in G1 plant organs, except G2 seed in each treatment, was *Gammaproteobacteria*, accounting for 48.5–92.8% of the ASVs abundance (Figure 6). The major difference between the microbiome distribution was contributed by class *Bacilli* in the G2 seeds of both treatments (G1 (T and C), which accounted for 78–83.3%, whilst in other organs, *Bacilli* only accounted for 0.2–2.4%. In G1 (T) plants, roots were the most microbially-diverse plant organs, accounting for 44 classes, followed by G2 seed, accounting for 37 classes. Shoot and leaf microbiomes comprised of 29 and 26 classes, respectively. In G1 (C), roots also dominated the microbiome with 43 classes followed by shoots consisting of 38 classes, with leaf and G2 seed accounting for 33 and 25 classes, respectively (Figure 6). The five most dominant bacterial classes were identified across different plant organs for each treatment. In the G1 (T) plants, the leaf was mainly dominated by *Gammaproteobacteria* (93%), “Unclassified Bacteria” (1.9%), *Alphaproteobacteria* (1.4%), *Actinobacteria* (1.3%) and *Verrucomicrobiae* (0.7%). The G1 (T) shoots contained *Gammaproteobacteria* (84.7%), “Unclassified Bacteria” (5.8%), *Bacilli* (2.3%), *Actinobacteria* (2.2%) and *Alphaproteobacteria* (1.9%). Whilst G1 (T) roots were comprised of *Gammaproteobacteria* (48.5%), *Alphaproteobacteria* (22.6%), *Verrucomicrobiae* (6.1%), *Actinobacteria* (4.7%) and *Bacteroidia* (3.9%). Comparatively, G2 (T) seed was dominated by *Bacilli* (83.3%), *Gammaproteobacteria* (11.2%), *Alphaproteobacteria* (1.6%), *Actinobacteria* (1.2%) and *Verrucomicrobiae* (0.6%) (Figure 6 and Supplementary Table S3). For G1 (C) plants, the leaves were mainly occupied by *Gammaproteobacteria* (83.2%), *Alphaproteobacteria* (5.3%), “Unclassified Bacteria” (4.8%), with *Bacilli* (1.5%) and *Actinobacteria* (1.5%) making up the balance. The distribution in G1 (C) shoots was *Gammaproteobacteria* (74.7%), “Unclassified Bacteria” (9.3%), *Alphaproteobacteria* (4.1%), *Actinobacteria* (3.8%) and *Bacilli* (2.5%); while in G1 (C) roots, *Gammaproteobacteria* (58.6%), *Alphaproteobacteria* (16.3%), *Clostridia* (5.7%), *Verrucomicrobiae* (4.6%) and *Actinobacteria* (2.9%) were prevalent. As mentioned previously, G2 (C) seed was dominated by *Bacilli* (78%), with the remainder of the class distribution being *Gammaproteobacteria*, (19.7%), *Actinobacteria* (1.3%), “Unclassified Bacteria” (0.3%) and *Alphaproteobacteria* (0.3%) (Figure 6 and Supplementary Table S3).

**Figure 7:**
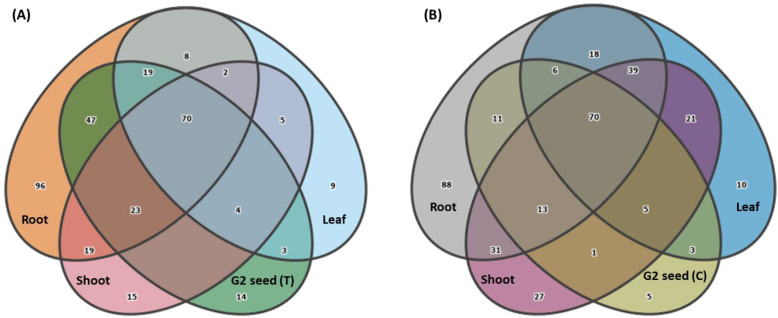
Venn diagrams showing the distribution of bacterial genera across G1 plant organs (root, shoot, leaf) and G2 seed for (**A**) Treatment G1 (T) and (**B**) Control, G1 (C).

The Venn diagrams displaying ASVs distributed by plant organs demonstrated that more unique genera were associated with the roots of G1 plants for both treatments with 96 genera in G1 (T) and 88 genera in G1 (C) plants. There was a reduced number of organ specific bacterial ASVs for upper plant organs, especially in the leaf (nine genera), shoot (15 genera) and G2 seed (14 genera) for G1 (T), and leaf (10 genera), shoot (27 genera) and G2 seed (five genera) for G1 (C) plants. There were 70 genera shared between all plant organs in both G1 (T) and G1 (C) (Figure 7). In total, 159 (88.3%) genera alone were shared between root and G2 (T) seed in commonality with shoot and leaf, while 100 genera (87.7%) were shared between root and G2 (C) seed in commonality with shoot and leaf. When considering only the bacteria genera shared between G2 seeds and each plant organ, the root-associated bacteria contributed most to the final G2 seed microbiome. There were 47 genera shared between G2 seed and roots for G1 (T) plants, whereas only 11 genera were shared between G2 seed and roots of G1 (C) plants. Interestingly, only a negligible number of genera were shared between G2 seeds and upper plant organs (shoot and leaf). Thus, only three genera were shared between leaf and seed in both G1 (T) and G1 (C), while no genera were shared between shoot and seed in G1 (T) plants (Figure 7).

### 3.6. Vertical Transmission of G1 Seed Microbiota across G1 Plant Organs and G2 Seed

**Figure 8:**
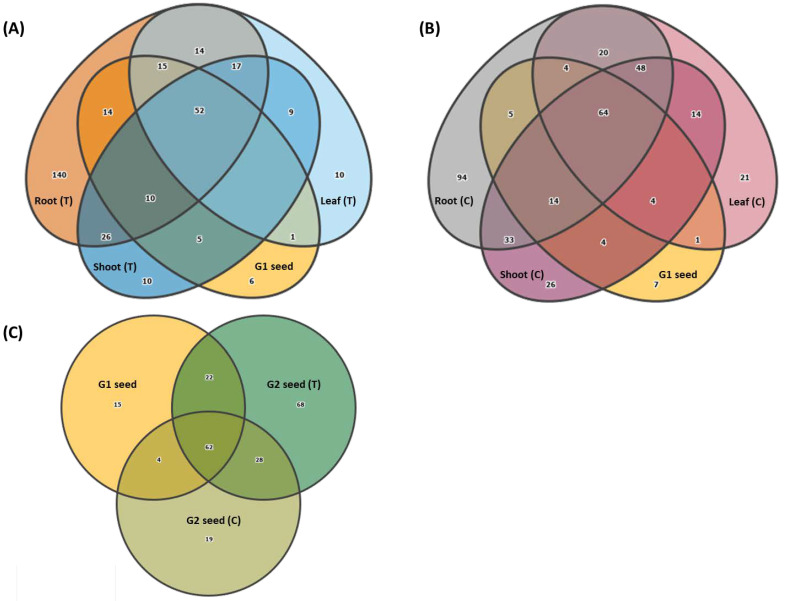
Venn diagrams showing the transmission of G1 bacterial genera across G1 plant organs (root, shoot and leaf) for(**A**) Treatment (T) and (**B**) Control and (**C**) G2 seed (T and C).

The Venn diagrams displaying G1 ASVs distributed across G1 plant organs demonstrated that both the above-ground and below ground G1 (T and C) organs were colonized by G1 seed microbiota. The G1 seed microbiota contributed largely to the plant microbiota and only six genera in G1 (T) and seven genera in G1 (C) were identified as unique to G1 seed (Figure 8A,B). For instance, in G1 (T) plants, 91 genera (88.3%) in root, 67 genera (65%) in shoot and 68 genera (66%) in leaf were present and in commonality with G1 seed (Figure 8A). Similarly, in G1 (C) plants, 87 genera (84.4%) in roots, 86 genera (83.4%) in shoots and 73 genera (70.8%) in leaf were present and in commonality with G1 seed (Figure 8B). Overall, there were 52 genera in G1 (T) and 64 genera in G1 (C) that were shared between root, shoot, leaf and G1 seed (Figure 8A,B). The influence of soil type on root bacterial diversity was clearly observed with 140 unique genera detected in G1 (T) plants (Figure 8A) whilst only 94 unique genera were associated with in G1 (C) roots (Figure 8B). Less unique genera were detected for upper plant organs with ten genera in the shoot and leaf of G1 (T) plants and 26 genera in shoot and 21 genera in the leaf of G1 (C) plants (Figure 8A,B). Interestingly, the importance of soil type in promoting vertical transmission of G1 seed microbiota to next generation seed was clearly identified with 90 genera (87.3%) in G2 (T) and 66 genera (64%) in G2 (C) being vertically transmitted to G2 seed (Figure 8C). Overall, there were 62 genera shared between G1 and G2 (T and C) seed. The G2 (T) seed microbiome was more diverse with 68 unique genera compared to G2 (C) with only 22 unique genera. The G1 seed consisted of 15 unique genera and shared 22 genera with G2 (T) and only four genera with G2 (C) seed (Figure 8C).

## 4. Discussion

Seed banks periodically must revive seeds into full plants to harvest their seeds to replenish and increase their own seed stocks to maintain seed viability for long-term sustainable storage, and also to supply accredited seed vaults with viable seeds for curation [23]. To our knowledge, there have been no published studies describing the effect of conventional seed bank practices on the composition of the seed microbiome of the second-generation (G2) or subsequent generations of seed, let alone if there is a noted difference in the seed microbiomes of subsequent generations of conventional, intermediate, or recalcitrant germinating seeds. However, more recent studies have started to explore similar lines of research [27,28]. Our study has demonstrated that growing *G. clandestina* seedings in potting mix supplemented with native bulk soil inoculum from the original plant source enhanced the seed bacterial transmissibility by 36.3% in G2 seed, suggesting that this approach can promote the conservation of the native seed microbiome during seed bulk generation. This, in turn, may contribute to the long-term viability of the seed in seed banks and thus increase seed germination and survivability.

### 4.1. G. clandestina Seed Microbiota Composition

In general, *G. clandestina* seed was mainly dominated by the microbial classes *Gammaproteobacteria, Alphaproteobacteria, Actinobacteria* and *Bacilli*, which is consistent with previous studies of the seed microbiome of the related domesticated crop *Glycine max* (soybean) [14,29]. Overall, the *G. clandestina* seed microbiota (G1 and G2) was primarily occupied by bacterial genera *Massilia, Pseudomonas*, *Paenibacillus*, *Cohnella*, *Pantoea* and *Uliginosibacterium*. Most of these genera have been reported as being associated with domesticated plant species including maize [30], soybean [31,32], wheat [33], cucumber [34], ryegrass [35], rice [36] as well as native alpine plants [21]. In this study, high variations in average abundance were observed for bacterial genera across G1 and G2 with rare taxa in the G1 seed becoming abundant in the G2 seed, while the dominant genera in G1 declined significantly in G2. For instance, *Massilia* (58.4%) and *Pseudomonas* (30.3%) dominated G1 seed, whereas the G2 seed microbiome was predominantly *Paenibacillus* with an average of 71.2% in G2 (T) and 73.6% in G2 (C). It has been postulated that the emergence of specific bacterial taxa, including rare taxa, can provide essential or new functions that can promote plant growth and nutrient cycling and can either provide an alternative or counterbalance functions that were missing in the abundant taxa of seed microbiomes [37,38,39]. The fact that the dominant genera in the G1 generation were not abundant in the G2 generation indicates that they may be less competitive in filling this required niche than the rare seed-borne taxa dominating the G2 seeds. If not so, this increase in abundance of specific ASVs could be associated to the life cycle effect, as it was demonstrated by Barret et al. [40] that emergence can shape the structure of seed microbiota. Bacteria belonging to the genus *Paenibacillus* were one of the dominating endophytic bacteria found in barley seed [41], wheat plants and seed that displayed beneficial attributes [10,42]. Previous studies determined that some *Paenibacillus* strains can enhance the seed germination rate due to their ability to produce cytokinins [43,44,45]. It was postulated by Goggin et al. [46] that a reduced concentration of bacterial cytokinins can result in a higher seed dormancy [46].

### 4.2. Effect of Soil Type on Composition and Vertical Transmission of Seed Microbiota

The data from this study indicated that there was clear a difference in seed microbiota composition based on soil type, with an increased number of low abundance taxa in the G2 (T) seed and a significant increase in the abundance of 11 genera. Previous studies demonstrated that the integration of two different ecosystems could result in the emergence of rare taxa as observed. Examples include the uneven mixing of two soil types of different physiochemical and bacterial compositions in soil microcosms [47], two soil types mixed to determine the assembly of rhizobia communities in root nodules [48], and on mixing of freshwater and marine water microbiomes [49]. Our results showed that the majority, but not all, of the G1 seed bacteria were vertically transmitted and make up a significant contribution to the *G. clandestina* plant microbiota. For instance, the below-ground and above-ground plant organs consisted of about 87–91% and 67–86% of G1-associated microbial communities, respectively. The seed-transmitted microbiota making up the majority of the plant microbiota has been reported on previously in other crop plants [28,50]. According to our metagenomic dataset, the larger subset of G1 seed microbiota occupied the root microbiota in both treatments (T and C), with a slightly higher proportion in G1 (T). These findings were further supported by a recent study that indicated that plant root-associated bacteria preferentially colonize their native host plant roots [51]. G1 microbes were also observed in above-ground organs, especially in shoots of G1 (C). This was in line with a study by Walsh et al. [50] that showed that the microbial communities of wheat (*Triticum aestivum*) seedlings were mainly derived from seed, although the plant microbiome composition was suggested to vary dependent on soil bacterial community composition. Interestingly, our results strongly indicated the influence of soil type on the assembly of G2 seed microbiota, whereby 25% more G1 seed microbes were vertically transmitted to G2 (T) seed compared to G2 (C) seed. Factors such as soil type, external environment, host genotype, dispersal agents, pollinators, and the floral microbiome have also been identified as potential drivers of the assembly and structure of seed microbiomes [52], and this study emphasises the essential role of soil type (i.e., native soil). The number of transmissible genera increased when plants were grown in potting mix supplemented with native soil, suggesting that the seed bacterial communities were recruited from both G1 seed and native soil [13]. Notably, this increase in vertical transmission was mainly related to more low abundance taxa being transferred into G2 (T) seed. The transmission of rare and intermediate bacterial genera from soil to seed has also been reported previously [4]. A review by Moran et al. [53] verified that the likelihood of intergenerational transmission of seed-associated endophytic bacteria is directly related to the indispensable function these microbes can confer to the plant. Similarly, the influence of soil microbiota on the assembly of plant microbiota has also been observed in pre-domesticated, ancient and modern varieties of maize [54].

Nevertheless, our study also showed that a significant amount of the G1 seed microbiota remains conserved across G2 (T and C) seed. In line with our findings, studies have detected a significant pool of conserved seed bacterial microbiota across plant generations in maize [55] and ryegrass [35] under glasshouse conditions. The seed transmissibility rate in our study may have been reduced, as seeds were first germinated on paper in sterile petri dishes before being planted in soil. Wolfgang et al. [39] postulated that the transmissibility of seed microbes could be underestimated due to the exposure of the plant roots to higher light and oxygen levels under laboratory conditions when compared to soil.

### 4.3. Effect of Soil Type on Redistribution of Bacterial Communities among Plant Organs

Overall, clear differences were observed between the bacterial profiles of the above-ground plant organs (shoot, leaf and G2 seed) and roots. These differences were mainly related to the presence or absence of low abundance classes and variations in the abundance of dominant bacterial classes. These results agree with previous studies in soybean [56], wheat [57] and *Arabidopsis* [58] which showed that above- and below-ground plant tissues were occupied by different microbial communities. In this study, PCoA plots showed separate clusters for “leaf and shoot” and “roots and seed”, as has been reported for the bacterial and fungal communities of *Populus* [59] and *G. max* [56] microbiomes. Notably, the root microbiota composition was more indicative of the soil type in which plants were grown, and this difference could be lower than expected, given that the native soil only represented around 10% of the soil mix in treatment G2 (T) and the fact that there was no adjustment time allowed for the soil-potting medium mixture. In a recent study, Samuel et al. [60] showed that the soil-root interface influenced the assembly of the endophytic bacterial community in the roots of rice plants. In our study, the interaction period between seedlings and native soil could have enabled the native soil microbiota to colonize the *G. clandestina* roots, and thereby increased the bacterial diversity of the *G. clandestina* root microbiome. The influence of media type on the composition of root microbiome has been reported for *Lolium perenne* [35], sugar beet [39], and barley [41] microbiomes. A recent study demonstrated that the soil bacterial diversity altered the physiochemical parameters (concentrations of NH_4_^+^, NO_3_^−^ and pH levels) of local soil, which subsequently guided the assembly of the plant microbiota composition, although the initial soil bacterial diversity was identified as the main driver of the seedling microbiota composition [4]. The results of our study have indicated a potential transmission of microbes from neighbouring plants via the aerosphere and physical contact with neighbouring plants, as no significant differences were observed between leaf and shoot microbiota across either G1 (T) or G1 (C) plants. The aerial dispersal of epiphytic bacteria by and from leaves of bean plants have been reported previously [61].

Our data showed that the largest subset of G2 (T) seed microbiota was recruited from roots compared to leaf and shoot. We found that 88.3% of G2 (T) bacteria originated from the roots, and by inference, the rhizosphere and rhizoplane surrounding the plant. Without native soil supplementation, 87.7% of genera from soil contributed to the G2 (C) microbiome. However, if we disregard the taxa that are common to all three (70 taxa), the proportion of taxa from the soil, through the root system, becomes 26.3% for G2 (C) seeds, and 49.4% with G2 (T) seeds, an almost 90% increase in the contribution of the soil microbiome to the next generation seed with soil treatment. Previous studies have suggested that the composition of the seed microbial communities reflects the microbial communities associated with roots [62,63]. This is the first time, to our knowledge, that recruitment of the root microbiome to the next generation seed has been demonstrated to be dependent on the soil the plant is growing in, therefore further studies exploring this area would strengthen our understanding about the role of native soil and the root microbiome in the assembly of the G2 seed microbiota.

Despite sharing the most genera, the dominant bacterial classes in seed, including *Bacilli*, were much less abundant in roots, which were mainly occupied by *Gammaproteobacteria*. At the genus level, this corresponded with a significant increased abundance of *Paenibacillus* in G2 seed. Yang et al. [41] in their study showed that the dominant bacteria associated with barley seed including *Enterobacteriaceae* and *Paenibacillaceae* become less abundant in roots when plants were grown in soil, suggesting an influence of soil microbial communities on the composition of the root microbiome. *Paenibacillus* belong to a group of phosphate-solubilizing bacteria [64]. In this study, the nutrient rich (NPK) environment of the commercial potting mix may have influenced the assembly of the seed microbiota, since NPK was not in deficit. It was established by Widdig et al. [65] that the addition of nitrogen and phosphorous altered the composition of phosphorous-solubilizing bacteria in grassland soils. Their findings showed a high abundance of bacterial genera including *Pseudomonas*, *Enterobacterales*, *Bacillus* and *Paenibacillus* in most soil samples. Interestingly, they also reported a lower abundance of *Pseudomonas* and an increased number of OTUs for *Enterobacterales* after the addition of N and NP. This is consistent, to some extent, with our results, as the abundance of *Pseudomonas* significantly declined, especially in G2 (T) seed, while the above-ground organs (shoot and leaf) were dominated by *Enterobacterales* (Supplementary Figure S2). In another study, Kang et al. [66] demonstrated that nitrogen fertilization was able to modulate the beneficial rhizosphere interactions in the cucumber plants, and suggested the rationalisation of the use of nitrogen fertilizers to promote beneficial microbial interactions. Other factors, such as the influence of the floral microbiome on the assembly and composition of seed microbiota, remain to be further investigated, although some studies indicate that this, too, has an influence on the seed microbiome assembly [67,68].

## 5. Conclusions

In conclusion, 16S rRNA profiling of *G. clandestina* plant organs grown with an initial inoculum of native soil, and seedlings derived from these plants, revealed the enhanced retention of generation one (G1) seed-borne bacteria compared to seedlings derived from plants solely grown in commercial potting mix. This was largely through vertical transmission of low abundance taxa that were present in the roots of the native soil-treated plants. Given that a mere 10% soil inoculum from the original plant soil did influence the assembly of seed microbiota, we suggest that replanting seed in the glasshouse with a larger ratio of local soil, or using native soil only, may improve the conservation of the seed microbiota from G1 to G2 and subsequent generations. A better understanding about the role of soil nutrient composition in plant microbiome assembly would be extremely helpful in crafting strategies to conserve native seed microbiota.

## Data Availability

All raw sequence derived from this experiment were submitted into the Short Read Archive of NCBI and can be found under the BioProject accession number PRJNA810761.

## Supplementary Materials

The Supplementary Material for this article can be found online at https://www.mdpi.com/article/10.3390/microorganisms10040750/s1, Table S1. Relative abundance of the bacterial classes associated with the G. clandestina seed microbiome belonging to G1 and G2 (T and C) seed. Taxa occurring at >0.1% are highlighted in bold. Table S2. Relative abundance of the bacterial genera associated with the G. clandestina seed microbiome belonging to G1 and G2 (T and C) seed. Taxa occurring at >0.1% are highlighted in bold. Table S3. Relative abundance of the bacterial classes associated with the G. clandestina microbiome belonging to G1 (T and C) plant organs (root, shoot and leaf) and G2 (T and C) seed. Taxa occurring at >0.1% are highlighted in bold. Table S4. Relative abundance of the bacterial genera associated with the G. clandestina belonging to G1 (T and C) plant organs (root, shoot, leaf and G2 seed. Taxa occurring at >0.1% are highlighted in bold. Table S5. P-values of the comparison between G. clandestina seed samples belonging to G1 and G2 (T and C) seed. Significant differences were determined using the Kruskal Wallis pairwise test using Alpha diversity (Observed features) and are highlighted in bold. Table S6. P-values of the comparison between G. clandestina seed samples belonging to G1 and G2 (T and C) seed. Significant differences were determined using the pairwise-ANOSIM test using Beta diversity (Jaccard distance) metrics and are highlighted in bold. Table S7. P-values of the comparison between G1 plant organs of G. clandestina belonging to root, shoot and leaf and G2 seed grown in two soil treatments (T and C). Significant differences were determined using the Kruskal Wallis pairwise test for Alpha diversity (Observed features) and are highlighted in bold. Table S8. P-values of the comparison between G1 plant organs of G. clandestina belonging to root, shoot and leaf and G2 seed grown in two soil treatments (T and C). Significant differences were determined using pairwise-ANOSIM test using Beta diversity (Jaccard distance) metrics and are highlighted in bold. Figure S1. G. clandestina seedlings at the unfolded cotyledon growth stage. Figure S2. Rarefaction curves showing the number of observed ASVs at a sampling depth of 1680 sequences when data was grouped by soil treatment for microbiome profiling of G. clandestina seed. Each coloured line represents one sample. Figure S3. Rarefaction curves showing the number of observed ASVs at a sampling depth of 1172 sequences when data was grouped by soil treatment for microbiome profiling of G. clandestina plant organs. Each coloured line represents one sample.
