# Supplementary material for: The Role of Soil Microbial Diversity in the Conservation of Native Seed Bacterial Microbiomes
Source: Microorganisms. 2022 Mar 30;10(4):750. doi: 10.3390/microorganisms10040750 (PMC9028870; doi:10.3390/microorganisms10040750)
Supplement: Supplementary file 1 [file microorganisms-10-00750-s001.zip › microorganisms-1632258-supplementary.pdf]

Supplementary materials

Table S1. Relative abundance of the bacterial classes associated with the *G. clandestina* seed microbiome *belonging* to G1 and G2 (T and C) seed. Taxa occurring at >0.1% are highlighted in bold.

Taxonomic Level		Relative Abundance (%)		
**Phylum**	**Class**	**G1 Seed**	**G2 Seed (T)**	**G2 Seed (C)**
Proteobacteria	*Alphaproteobacteria*	**1.830**	**1.617**	**0.208**
Actinobacteriota	*Actinobacteria*	**1.900**	**1.152**	**1.341**
Verrucomicrobiota	*Verrucomicrobiae*	**0.327**	**0.587**	0.098
Proteobacteria	*Gammaproteobacteria*	**93.904**	**11.486**	**19.894**
Bacteroidota	*Bacteroidia*	**0.734**	**0.319**	0.055
Elusimicrobiota	*Elusimicrobia*	0.040	0.023	0.007
Armatimonadota	*Fimbriimonadia*	0.010	0.010	0.000
Acidobacteriota	*Acidobacteriae*	0.079	0.086	0.025
Firmicutes	*Bacilli*	**0.119**	**83.566**	**78.014**
Unclassified_Bacteria		**0.496**	**0.463**	**0.273**
Planctomycetota	*Phycisphaerae*	0.050	0.076	0.007
Actinobacteriota	*Thermoleophilia*	0.074	0.040	0.003
Bdellovibrionota	*Oligoflexia*	0.000	0.002	0.000
Myxococcota	*Polyangia*	0.000	0.002	0.003
Patescibacteria	*Saccharimonadia*	0.089	**0.132**	0.013
Acidobacteriota	*Vicinamibacteria*	0.035	0.043	0.000
Cyanobacteria	*Vampirivibrionia*	0.000	0.007	0.003
Actinobacteriota	*Acidimicrobiia*	0.000	0.003	0.000
Myxococcota	*Myxococcia*	0.015	0.008	0.000
Firmicutes	*Clostridia*	0.005	0.041	0.003

Spirochaetota	*Spirochaetia*	0.050	0.063	0.002
Patescibacteria	*Parcubacteria*	0.010	0.005	0.000
Gemmatimonadota	*Gemmatimonadetes*	0.020	0.030	0.000
Bdellovibrionota	*Bdellovibrionia*	0.015	0.010	0.000
Planctomycetota	*Planctomycetes*	0.025	0.084	0.012
FCPU426	*FCPU426*	0.089	0.056	0.015
Chloroflexi	*Ktedonobacteria*	0.084	0.081	0.021
Bacteroidota	*Ignavibacteria*	0.000	0.003	0.000
Firmicutes	*Negativicutes*	0.000	0.003	0.003

Table S2. Relative abundance of the bacterial genera associated with the *G. clandestina* seed microbiome *belonging* to G1 and G2 (T and C) seed. Taxa occurring at >0.1% are highlighted in bold.

**Taxonomic Level**			**Relative Abundance (%)**		
**Phylum**	**Class**	**Order / Family / Genus**	**G1 Seed**	**G2 Seed (T)**	**G2 Seed (C)**
Firmicutes	*Bacilli*	*Paenibacillus*	**0.119**	**71.288**	**73.679**
Proteobacteria	*Gammaproteobacteria*	*Massilia*	**58.497**	0.017	0.013
Proteobacteria	*Gammaproteobacteria*	*Pseudomonas*	**30.372**	**0.364**	**14.831**
Firmicutes	*Bacilli*	*Cohnella*	0.000	**9.342**	**3.193**
Proteobacteria	*Gammaproteobacteria*	*Pantoea*	**0.174**	**4.468**	**3.937**
Proteobacteria	*Gammaproteobacteria*	*Uliginosibacterium*	**2.133**	**3.257**	**0.522**
Firmicutes	*Bacilli*	*Bacillus*	0.000	**2.889**	**1.126**
Proteobacteria	*Gammaproteobacteria*	*Comamonadaceae* (*F*)	**1.533**	**1.144**	**0.208**
Actinobacteriota	*Actinobacteria*	*Nocardioides*	0.035	0.005	**1.186**
Actinobacteriota	*Actinobacteria*	*Curtobacterium*	**1.086**	0.003	0.000
Actinobacteriota	*Actinobacteria*	*Streptomyces*	**0.595**	**0.873**	**0.104**
Proteobacteria	*Gammaproteobacteria*	*Burkholderia-Caballeronia-Paraburkholderia*	**0.357**	**0.832**	**0.103**
Proteobacteria	*Alphaproteobacteria*	*Sphingomonas*	**0.809**	0.000	0.000
Proteobacteria	*Gammaproteobacteria*	*Rhodanobacter*	**0.486**	**0.678**	0.094
Bacteroidota	*Bacteroidia*	*Hymenobacter*	**0.585**	0.000	0.000
Verrucomicrobiota	*Verrucomicrobiae*	*Uncultured Pedosphaerales*	**0.283**	**0.501**	0.086
Unclassified__Bacteria			**0.496**	**0.463**	**0.273**
Proteobacteria	*Alphaproteobacteria*	*Asticcacaulis*	**0.313**	**0.270**	0.031
Proteobacteria	*Alphaproteobacteria*	*Uncultured Micropepsales*	**0.134**	**0.215**	0.036
Actinobacteriota	*Actinobacteria*	*Actinoplanes*	**0.104**	**0.179**	0.033

Proteobacteria	*Alphaproteobacteria*	*Devosia*	0.089	**0.175**	0.017
Proteobacteria	*Alphaproteobacteria*	*Pseudolabrys*	0.020	**0.155**	0.012
Proteobacteria	*Gammaproteobacteria*	*Enterobacterales* (*O*)	0.045	**0.137**	0.031
Proteobacteria	*Alphaproteobacteria*	*Hephaestia*	0.025	**0.136**	0.012
Bacteroidota	*Bacteroidia*	*Mucilaginibacter*	0.094	**0.114**	0.017
Proteobacteria	*Gammaproteobacteria*	*Thauera*	0.010	**0.103**	0.012
Proteobacteria	*Alphaproteobacteria*	*Xanthobacteraceae* (*F*)	0.055	0.099	0.021
Proteobacteria	*Alphaproteobacteria*	*Dongia*	0.035	0.094	0.013
Proteobacteria	*Gammaproteobacteria*	*Chujaibacter*	0.030	0.093	0.008
Proteobacteria	*Gammaproteobacteria*	*Bordetella*	0.030	0.091	0.020
FCPU426	*FCPU426*	*FCPU426 (F)*	0.089	0.056	0.015
Proteobacteria	*Alphaproteobacteria*	*Uncultured Elsterales*	0.005	0.088	0.010
Patescibacteria	*Saccharimonadia*	*LWQ8 (F)*	0.079	0.045	0.005
Proteobacteria	*Gammaproteobacteria*	*Acidibacter*	0.055	0.076	0.017
Proteobacteria	*Alphaproteobacteria*	*Hyphomicrobium*	0.060	0.071	0.005
Chloroflexi	*Ktedonobacteria*	*Uncultured Ktedonobacterales*	0.064	0.068	0.018
Planctomycetota	*Phycisphaerae*	*CPla-3_termite_group*	0.050	0.064	0.007
Spirochaetota	*Spirochaetia*	*Spirochaeta_2*	0.050	0.063	0.002
Verrucomicrobiota	*Verrucomicrobiae*	*Opitutus*	0.020	0.063	0.003
Proteobacteria	*Alphaproteobacteria*	*Methylobacterium-Methylorubrum*	0.055	0.000	0.000
Proteobacteria	*Alphaproteobacteria*	*Uncultured Rhodospirillales*	0.055	0.008	0.003
Proteobacteria	*Gammaproteobacteria*	*TRA3-20 (F)*	0.055	0.013	0.008
Planctomycetota	*Planctomycetes*	*Uncultured Gemmatales*	0.010	0.048	0.002
Proteobacteria	*Gammaproteobacteria*	*Oxalobacteraceae* (*F*)	0.040	0.043	0.010
Acidobacteriota	*Vicinamibacteria*	*Uncultured Vicinamibacterales*	0.035	0.043	0.000
Bacteroidota	*Bacteroidia*	*Uncultured Chitinophagales*	0.015	0.043	0.013
Firmicutes	*Clostridia*	*Clostridium_sensu_stricto_1*	0.005	0.041	0.003
Elusimicrobiota	*Elusimicrobia*	*Lineage_IV (F)*	0.040	0.023	0.007
Actinobacteriota	*Thermoleophilia*	*67-14 (F)*	0.040	0.005	0.000

Bacteroidota	*Bacteroidia*	*Uncultured Cytophagales*	0.000	0.040	0.000
Actinobacteriota	*Thermoleophilia*	*Uncultured Gaiellales*	0.035	0.018	0.003
Acidobacteriota	*Acidobacteriae*	*Bryobacter*	0.035	0.012	0.010
Proteobacteria	*Alphaproteobacteria*	*Phenylobacterium*	0.020	0.033	0.010
Patescibacteria	*Saccharimonadia*	*Saccharimonadales* (*O*)	0.000	0.033	0.000
Proteobacteria	*Alphaproteobacteria*	*Mesorhizobium*	0.010	0.031	0.002
Proteobacteria	*Alphaproteobacteria*	*Bradyrhizobium*	0.030	0.018	0.000
Patescibacteria	*Saccharimonadia*	*Saccharimonadales*	0.010	0.030	0.008
Proteobacteria	*Alphaproteobacteria*	*Azospirillum*	0.010	0.028	0.002
Proteobacteria	*Alphaproteobacteria*	*Novosphingobium*	0.000	0.028	0.003
Proteobacteria	*Gammaproteobacteria*	*MND1*	0.010	0.026	0.003
Actinobacteriota	*Actinobacteria*	*Rothia*	0.005	0.026	0.012
Proteobacteria	*Alphaproteobacteria*	*Sphingomonadaceae* (*F*)	0.025	0.017	0.003
Proteobacteria	*Gammaproteobacteria*	*KF-JG30-C25 (F)*	0.025	0.015	0.010
Acidobacteriota	*Acidobacteriae*	*Subgroup_13*	0.025	0.005	0.000
Actinobacteriota	*Actinobacteria*	*Microbacterium*	0.025	0.002	0.000
Bacteroidota	*Bacteroidia*	*env.OPS_17 (F)*	0.025	0.023	0.000
Proteobacteria	*Alphaproteobacteria*	*Caulobacteraceae* (*F*)	0.015	0.025	0.000
Proteobacteria	*Gammaproteobacteria*	*Dokdonella*	0.000	0.025	0.002
Bacteroidota	*Bacteroidia*	*Taibaiella*	0.000	0.025	0.000
Proteobacteria	*Alphaproteobacteria*	*Uncultured Rhodospirillales*	0.000	0.025	0.000
Gemmatimonadota	*Gemmatimonadetes*	*Gemmatimonas*	0.000	0.025	0.000
Patescibacteria	*Saccharimonadia*	*WWH38 (F)*	0.000	0.025	0.000
Actinobacteriota	*Actinobacteria*	*Acidothermus*	0.005	0.021	0.000
Proteobacteria	*Gammaproteobacteria*	*SC-I-84 (F)*	0.000	0.021	0.000
Proteobacteria	*Gammaproteobacteria*	*Enterobacteriaceae* (*F*)	0.000	0.002	0.021
Proteobacteria	*Alphaproteobacteria*	*Micropepsis*	0.020	0.000	0.002
Actinobacteriota	*Actinobacteria*	*Cellulomonas*	0.020	0.013	0.002
Gemmatimonadota	*Gemmatimonadetes*	*Uncultured Gemmatimonadales*	0.020	0.005	0.000

Proteobacteria	*Alphaproteobacteria*	*Parvibaculum*	0.020	0.000	0.003
Firmicutes	*Bacilli*	*Pullulanibacillus*	0.000	0.020	0.000
Bacteroidota	*Bacteroidia*	*Sphingobacteriaceae* (*F*)	0.000	0.020	0.005
Proteobacteria	*Gammaproteobacteria*	*Steroidobacter*	0.010	0.018	0.013
Firmicutes	*Bacilli*	*Bacillales* (O)	0.000	0.018	0.000
Bacteroidota	*Bacteroidia*	*Wandonia*	0.015	0.017	0.005
Acidobacteriota	*Acidobacteriae*	*Occallatibacter*	0.005	0.017	0.002
Verrucomicrobiota	*Verrucomicrobiae*	*Uncultured Opitutales*	0.005	0.017	0.005
Proteobacteria	*Alphaproteobacteria*	*D05-2 (F)*	0.000	0.017	0.002
Actinobacteriota	*Thermoleophilia*	*Conexibacter*	0.000	0.017	0.000
Proteobacteria	*Gammaproteobacteria*		0.015	0.008	0.003
Myxococcota	*Myxococcia*	*Anaeromyxobacter*	0.015	0.008	0.000
Bdellovibrionota	*Bdellovibrionia*	*Bdellovibrio*	0.015	0.010	0.000
Planctomycetota	*Planctomycetes*	*Uncultured Pirellulales*	0.015	0.010	0.010
Actinobacteriota	*Actinobacteria*	*Parafrigoribacterium*	0.005	0.015	0.000
Acidobacteriota	*Acidobacteriae*	*Acidobacteriaceae_(Subgroup_1)* (F)	0.005	0.015	0.003
Proteobacteria	*Gammaproteobacteria*	*JG36-TzT-191 (F)*	0.000	0.015	0.000
Planctomycetota	*Planctomycetes*	*Aquisphaera*	0.000	0.015	0.000
Acidobacteriota	*Acidobacteriae*	*Subgroup_2*	0.010	0.013	0.000
Proteobacteria	*Alphaproteobacteria*	*Uncultured Acetobacterales*	0.000	0.013	0.003
Bacteroidota	*Bacteroidia*	*Arachidicoccus*	0.000	0.013	0.013
Proteobacteria	*Alphaproteobacteria*	*Micropepsaceae* (F)	0.000	0.013	0.003
Proteobacteria	*Gammaproteobacteria*	*Castellaniella*	0.000	0.008	0.013
Actinobacteriota	*Actinobacteria*	*Cutibacterium*	0.010	0.012	0.005
Proteobacteria	*Alphaproteobacteria*	*Acetobacteraceae* (F)	0.000	0.012	0.000
Planctomycetota	*Phycisphaerae*	*WD2101_soil_group*	0.000	0.012	0.000
Planctomycetota	*Planctomycetes*	*Uncultured Planctomycetales*	0.000	0.012	0.000
Proteobacteria	*Alphaproteobacteria*	*Devosiaceae* (F)	0.000	0.012	0.003
Acidobacteriota	*Acidobacteriae*	*Uncultured Acidobacteriales*	0.000	0.012	0.005

Armatimonadota	*Fimbriimonadia*	*Fimbriimonadaceae*	0.010	0.010	0.000
Proteobacteria	*Alphaproteobacteria*	*Candidatus_Finniella*	0.010	0.000	0.000
Patescibacteria	*Parcubacteria*	*Candidatus_Nomurabacteria*	0.010	0.005	0.000
Proteobacteria	*Alphaproteobacteria*	*Rhizomicrobium*	0.010	0.008	0.002
Verrucomicrobiota	*Verrucomicrobiae*	*Candidatus_Udaeobacter*	0.010	0.002	0.003
Proteobacteria	*Gammaproteobacteria*	*Yersiniaceae* (F)	0.010	0.000	0.002
Chloroflexi	*Ktedonobacteria*	*FCPS473*	0.010	0.003	0.000
Proteobacteria	*Gammaproteobacteria*	*Escherichia-Shigella*	0.010	0.005	0.000
Bacteroidota	*Bacteroidia*	*Puia*	0.000	0.008	0.000
Firmicutes	*Bacilli*		0.000	0.000	0.008
Bacteroidota	*Bacteroidia*	*Sporocytophaga*	0.000	0.008	0.002
Chloroflexi	*Ktedonobacteria*	*JG30-KF-AS9 (F)*	0.000	0.008	0.000
Cyanobacteria	*Vampirivibrionia*	*Obscuribacteraceae*	0.000	0.007	0.003
Bacteroidota	*Bacteroidia*	*Cytophagales* (O)	0.000	0.007	0.000
Proteobacteria	*Gammaproteobacteria*	*Acinetobacter*	0.000	0.007	0.003
Proteobacteria	*Alphaproteobacteria*	*Sandaracinobacter*	0.000	0.005	0.007
Proteobacteria	*Gammaproteobacteria*	*Uncultured Gammaproteobacteria*	0.000	0.007	0.000
Firmicutes	*Bacilli*	*Ammoniphilus*	0.000	0.007	0.002
Proteobacteria	*Gammaproteobacteria*	*Candidimonas*	0.005	0.003	0.002
Proteobacteria	*Gammaproteobacteria*	*Dyella*	0.005	0.000	0.000
Proteobacteria	*Alphaproteobacteria*	*Methylovirgula*	0.005	0.005	0.002
Proteobacteria	*Alphaproteobacteria*	*Uncultured Alphaproteobacteria*	0.005	0.003	0.000
Verrucomicrobiota	*Verrucomicrobiae*	*Ellin516*	0.005	0.000	0.000
Chloroflexi	*Ktedonobacteria*	*C0119 (F)*	0.005	0.002	0.003
Verrucomicrobiota	*Verrucomicrobiae*	*Prosthecobacter*	0.005	0.000	0.000
Chloroflexi	*Ktedonobacteria*	*Ktedonobacteraceae* (F)	0.005	0.000	0.000
Actinobacteriota	*Actinobacteria*	*Frankiales* (O)	0.005	0.000	0.000
Actinobacteriota	*Actinobacteria*	*Luedemannella*	0.005	0.000	0.000
Acidobacteriota	*Acidobacteriae*	*Acidobacterium*	0.000	0.005	0.000

Proteobacteria	*Gammaproteobacteria*	*Ellin6067*	0.000	0.005	0.000
Verrucomicrobiota	*Verrucomicrobiae*	*Lacunisphaera*	0.000	0.003	0.000
Firmicutes	*Bacilli*	*Anaerobacillus*	0.000	0.003	0.000
Proteobacteria	*Alphaproteobacteria*	*Uncultured Micavibrionales*	0.000	0.003	0.002
Acidobacteriota	*Acidobacteriae*	*Acidipila*	0.000	0.003	0.003
Actinobacteriota	*Acidimicrobiia*	*IMCC26256 (F)*	0.000	0.003	0.000
Firmicutes	*Bacilli*	*Streptococcus*	0.000	0.000	0.003
Firmicutes	*Bacilli*	*Staphylococcus*	0.000	0.000	0.003
Proteobacteria	*Alphaproteobacteria*	*Rhizobiaceae* (F)	0.000	0.003	0.000
Proteobacteria	*Gammaproteobacteria*	*Halomonas*	0.000	0.003	0.003
Proteobacteria	*Gammaproteobacteria*	*Methylophilus*	0.000	0.003	0.000
Bacteroidota	*Ignavibacteria*	*UA-50 (F)*	0.000	0.003	0.000
Firmicutes	*Negativicutes*	*Anaerosinus*	0.000	0.003	0.003
Actinobacteriota	*Actinobacteria*	*Actinospica*	0.000	0.003	0.000
Acidobacteriota	*Acidobacteriae*	*Granulicella*	0.000	0.003	0.002
Proteobacteria	*Alphaproteobacteria*		0.000	0.002	0.000
Bdellovibrionota	*Oligoflexia*	*0319-6G20 (F)*	0.000	0.002	0.000
Myxococcota	*Polyangia*	*BIrii41 (F)*	0.000	0.000	0.002
Acidobacteriota	*Acidobacteriae*	*Candidatus_Solibacter*	0.000	0.002	0.000
Proteobacteria	*Alphaproteobacteria*	*Methyloligella*	0.000	0.002	0.000
Proteobacteria	*Gammaproteobacteria*	*Candidatus_Portiera*	0.000	0.000	0.002
Myxococcota	*Polyangia*	*Haliangium*	0.000	0.002	0.002
Proteobacteria	*Gammaproteobacteria*	*Uncultured Burkholderiales*	0.000	0.000	0.002
Proteobacteria	*Alphaproteobacteria*	*Uncultured Rhodospirillales*	0.000	0.002	0.000
Proteobacteria	*Alphaproteobacteria*	*SWB02*	0.000	0.002	0.000
Verrucomicrobiota	*Verrucomicrobiae*	*Opitutaceae* (F)	0.000	0.002	0.000
Bacteroidota	*Bacteroidia*	*CWT_CU03-E12 (F)*	0.000	0.002	0.000

Table S3. Relative abundance of the bacterial classes associated with the *G. clandestina* microbiome belonging to G1 (T and C) plant organs (root, shoot and leaf) and G2 (T and C) seed. Taxa occurring at >0.1% are highlighted in bold.

**Taxonomic Level**		**Relative Abundance (%)**							
**Phylum**	**Class**	**G2 Seed (T)**	**G2 Seed (C)**	**Leaf (T)**	**Leaf (C)**	**Shoot (T)**	**Shoot (C)**	**Root (T)**	**Root (C)**
Proteobacteria	*Alphaproteobacteria*	**1.690**	**0.263**	**1.493**	**5.349**	**1.924**	**4.124**	**22.661**	**16.276**
Unidentified__Bacteria		**0.448**	**0.341**	**1.948**	**4.818**	**5.849**	**9.279**	**0.441**	**0.164**
Proteobacteria	*Gammaproteobacteria*	**11.204**	**19.743**	**92.833**	**83.270**	**84.742**	**74.673**	**48.521**	**58.589**
Actinobacteriota	*Actinobacteria*	**1.204**	**1.320**	**1.337**	**1.483**	**2.180**	**3.797**	**4.693**	**2.986**
Verrucomicrobiota	*Verrucomicrobiae*	**0.642**	**0.128**	**0.747**	**1.030**	**0.962**	**1.699**	**6.172**	**4.636**
Firmicutes	*Bacilli*	**83.385**	**77.972**	**0.555**	**1.569**	**2.257**	**2.460**	**0.164**	**0.384**
Bacteroidota	*Bacteroidia*	**0.339**	0.052	**0.327**	**0.656**	**0.349**	**0.647**	**3.875**	**2.467**
Planctomycetota	*Planctomycetes*	**0.111**	0.005	0.028	**0.118**	**0.101**	**0.348**	**0.476**	**0.334**
Patescibacteria	*Saccharimonadia*	**0.187**	0.031	0.078	**0.643**	**0.535**	**0.505**	**2.510**	**1.379**
Acidobacteriota	*Acidobacteriae*	0.097	0.024	**0.128**	**0.171**	**0.202**	**0.292**	**1.920**	**1.977**
Chloroflexi	*Ktedonobacteria*	0.083	0.017	0.085	**0.138**	**0.132**	**0.334**	**1.479**	**0.206**
Actinobacteriota	*Thermoleophilia*	0.085	0.021	0.050	**0.105**	**0.178**	**0.235**	**0.960**	**0.533**
Firmicutes	*Clostridia*	0.052	0.005	0.014	0.098	**0.109**	**0.178**	**1.515**	**5.703**
FCPU426	*FCPU426*	0.066	0.014	0.100	0.072	**0.155**	**0.114**	**0.292**	**0.306**
Spirochaetota	*Spirochaetia*	0.071	0.000	0.043	0.026	0.008	**0.114**	**0.171**	**0.171**
Gemmatimonadota	*Gemmatimonadetes*	0.052	0.000	0.021	**0.125**	0.000	0.050	**0.277**	**0.228**
Planctomycetota	*Phycisphaerae*	**0.111**	0.007	0.007	0.092	0.031	0.078	**0.228**	**0.135**

Elusimicrobiota	*Elusimicrobia*	0.017	0.009	0.092	0.000	0.016	0.085	**0.412**	**0.299**
Armatimonadota	*Fimbriimonadia*	0.007	0.002	0.007	0.020	0.016	0.021	**0.107**	**0.128**
Cyanobacteria	*Vampirivibrionia*	0.014	0.012	0.007	0.046	0.039	0.014	**0.149**	**0.185**
Firmicutes	*Negativicutes*	0.002	0.002	0.000	0.000	0.000	0.036	**1.763**	**0.284**
Desulfobacterota	*Desulfuromonadia*	0.000	0.005	0.000	0.033	0.000	0.021	**0.235**	**0.867**
Acidobacteriota	*Vicinamibacteria*	0.036	0.000	0.021	0.020	0.008	0.057	**0.142**	0.064
Dependentiae	*Babeliae*	0.005	0.000	0.000	0.000	0.000	0.000	**0.121**	0.028
Bdellovibrionota	*Bdellovibrionia*	0.014	0.005	0.000	0.000	0.016	0.014	**0.149**	0.085
Campilobacterota	*Campylobacteria*	0.000	0.000	0.000	0.007	0.000	0.000	0.000	**0.455**
Actinobacteriota	*Acidimicrobiia*	0.007	0.000	0.000	0.026	0.031	0.064	0.064	**0.128**
Firmicutes	*Desulfitobacteriia*	0.002	0.000	0.000	0.000	0.016	0.014	0.021	**0.739**
Deinococcota	*Deinococci*	0.000	0.000	0.000	0.007	0.054	**0.327**	0.000	0.000
Cyanobacteria	*Cyanobacteriia*	0.000	0.000	0.000	0.000	0.016	**0.263**	0.000	0.000
Bdellovibrionota	*Oligoflexia*	0.009	0.000	0.000	0.007	0.000	0.007	0.021	0.014
Armatimonadota	*Armatimonadia*	0.002	0.000	0.000	0.000	0.000	0.000	0.007	0.014
Chloroflexi	*Unidentified__Chloroflexi*	0.000	0.000	0.000	0.007	0.000	0.007	0.000	0.000
Chloroflexi	*Chloroflexia*	0.000	0.002	0.000	0.000	0.000	0.000	0.021	0.057
Verrucomicrobiota	*Lentisphaeria*	0.000	0.000	0.000	0.000	0.000	0.000	0.014	0.000
Chloroflexi	*Dehalococcoidia*	0.002	0.005	0.000	0.007	0.000	0.007	0.007	0.000
Verrucomicrobiota	*Chlamydiae*	0.005	0.000	0.000	0.007	0.008	0.000	0.092	0.007
Proteobacteria	*Unidentified__Proteobacteria*	0.000	0.000	0.000	0.000	0.000	0.000	0.043	0.021
Myxococcota	*Polyangia*	0.000	0.007	0.014	0.007	0.023	0.014	0.028	0.000
Chloroflexi	*Kd4-96*	0.007	0.000	0.000	0.000	0.000	0.000	0.057	0.028
Firmicutes	*Sulfobacillia*	0.000	0.000	0.000	0.007	0.008	0.000	0.000	0.028
Verrucomicrobiota	*Omnitrophia*	0.000	0.000	0.000	0.000	0.000	0.000	0.000	0.007
Chloroflexi	*Tk10*	0.000	0.000	0.000	0.000	0.000	0.000	0.014	0.000
WPS-2	*Wps-2*	0.000	0.000	0.007	0.000	0.000	0.000	0.064	0.021
Myxococcota	*Myxococcia*	0.012	0.007	0.000	0.007	0.039	0.014	0.028	0.007

Abditibacteriota	*Abditibacteria*	0.000	0.000	0.000	0.000	0.000	0.000	0.000	0.014
Patescibacteria	*Parcubacteria*	0.009	0.000	0.000	0.020	0.000	0.000	0.014	0.021
Chloroflexi	*Jg30-Kf-Cm66*	0.000	0.000	0.028	0.000	0.000	0.000	0.000	0.007
Firmicutes	*Unidentified__Firmicutes*	0.000	0.000	0.000	0.000	0.000	0.064	0.014	0.000
Firmicutes	*Thermaerobacteria*	0.000	0.000	0.000	0.000	0.000	0.007	0.000	0.000
Cyanobacteria	*Sericytochromatia*	0.000	0.000	0.021	0.000	0.000	0.000	0.021	0.007
Firmicutes	*Desulfotomaculia*	0.000	0.000	0.000	0.000	0.000	0.014	0.000	0.000
Firmicutes	*Thermoanaerobacteria*	0.000	0.000	0.000	0.000	0.000	0.000	0.007	0.000
RCP2-54	*Rcp2-54*	0.002	0.000	0.000	0.000	0.000	0.000	0.000	0.000
Chloroflexi	*Olb14*	0.002	0.000	0.000	0.000	0.000	0.007	0.028	0.007
Bacteroidota	*Ignavibacteria*	0.009	0.000	0.000	0.013	0.000	0.014	0.000	0.000
Desulfobacterota	*Uncultured*	0.005	0.000	0.007	0.000	0.000	0.000	0.000	0.000

Table S4. Relative abundance of the bacterial genera associated with the *G. clandestina* belonging to G1 (T and C) plant organs (root, shoot, leaf and G2 seed. Taxa occurring at >0.1% are highlighted in bold.

Taxonomic Level			Relative Abundance							
**Phylum**	**Class**	**Order / Family / Genus**	**G2 Seed (T)**	**G2 Seed (C)**	**Leaf (T)**	**Leaf (C)**	**Shoot (T)**	**Shoot (C)**	**Root (T)**	**Root (C)**
Proteobacteria	*Alphaproteobacteria*		0.009	0.000	0.014	0.007	0.023	0.014	0.078	0.007
Unclassified__Bacteria			**0.448**	**0.341**	**1.948**	**4.818**	**5.849**	**9.279**	**0.441**	**0.164**
Bdellovibrionota	*Oligoflexia*	*Silvanigrella*	0.000	0.000	0.000	0.000	0.000	0.000	0.007	0.000
Proteobacteria	*Gammaproteobacteria*	*Bordetella*	0.085	0.005	0.064	**0.236**	**0.109**	**0.235**	**1.493**	**4.721**
Planctomycetota	*Planctomycetes*	*Uncultured Pirellulaceae*	0.012	0.005	0.000	0.026	0.085	0.028	**0.220**	**0.107**
Actinobacteriota	*Actinobacteria*	*Nocardioides*	0.007	**1.152**	0.014	0.020	0.070	**0.242**	**0.299**	**0.213**
Proteobacteria	*Gammaproteobacteria*		**1.026**	**0.213**	**1.031**	**1.496**	**1.582**	**2.517**	**5.454**	**8.099**
Proteobacteria	*Alphaproteobacteria*	*Uncultured Elsterales*	0.073	0.012	**0.121**	**0.158**	0.062	**0.142**	**1.166**	**0.832**
Acidobacteriota	*Vicinamibacteria*		0.002	0.000	0.000	0.000	0.008	0.000	0.007	0.000
Proteobacteria	*Gammaproteobacteria*	*Pantoea*	**4.354**	**3.771**	**13.147**	**18.259**	**25.861**	**24.353**	**0.562**	**1.308**
Proteobacteria	*Alphaproteobacteria*	*Uncultured Rhodospirillales*	0.007	0.005	0.007	0.020	0.054	0.064	**0.604**	**0.306**
Actinobacteriota	*Actinobacteria*	*Acidothermus*	0.026	0.000	0.000	0.046	0.016	0.043	**0.825**	**0.853**
Actinobacteriota	*Actinobacteria*	*Parafrigoribacterium*	0.007	0.000	0.021	0.007	0.000	0.014	0.036	0.014
Verrucomicrobiota	*Verrucomicrobiae*	*Uncultured Pedosphaeraceae*	**0.533**	**0.109**	**0.512**	**0.886**	**0.931**	**1.315**	**5.468**	**3.989**
Planctomycetota	*Phycisphaerae*	*Uncultured Phycisphaeraceae*	0.000	0.000	0.000	0.000	0.000	0.000	0.007	0.000
Firmicutes	*Clostridia*	*Ruminiclostridium*	0.000	0.000	0.000	0.000	0.000	0.000	0.014	0.036
Actinobacteriota	*Actinobacteria*	*Uncultured Frankiales*	0.000	0.000	0.000	0.000	0.000	0.007	0.043	0.028

Acidobacteriota	*Acidobacteriae*	*Uncultured Acidobacteriaceae_(Subgroup_1)*	0.026	0.000	0.021	0.007	0.000	0.021	**0.178**	**0.270**
Proteobacteria	*Gammaproteobacteria*	*Candidimonas*	0.002	0.000	0.028	0.033	0.000	0.007	0.036	0.092
Dependentiae	*Babeliae*	*Vermiphilaceae (F)*	0.000	0.000	0.000	0.000	0.000	0.000	0.064	0.021
Proteobacteria	*Gammaproteobacteria*	*Nevskia*	0.000	0.000	0.000	0.026	0.016	0.021	0.000	0.021
Proteobacteria	*Alphaproteobacteria*	*SM2D12 (F)*	0.000	0.005	0.000	0.020	0.000	0.050	0.036	0.021
Acidobacteriota	*Acidobacteriae*	*Bryobacter*	0.012	0.000	0.007	0.026	0.016	0.036	**0.263**	**0.270**
Acidobacteriota	*Acidobacteriae*	*Acidipila*	0.014	0.007	0.021	0.053	0.054	0.036	**0.334**	**0.356**
Planctomycetota	*Planctomycetes*	*Schlesneria*	0.009	0.000	0.000	0.000	0.000	0.000	0.000	0.000
Proteobacteria	*Alphaproteobacteria*	*Uncultured Alphaproteobacteria*	0.007	0.007	0.007	0.007	0.023	0.000	**0.114**	**0.128**
Armatimonadota	*Armatimonadia*	*Armatimonadales (O)*	0.002	0.000	0.000	0.000	0.000	0.000	0.007	0.014
Actinobacteriota	*Actinobacteria*	*Actinomadura*	0.000	0.000	0.000	0.000	0.000	0.000	0.014	0.000
Chloroflexi	*Ktedonobacteria*	*JG30-KF-AS9 (F)*	0.014	0.000	0.000	0.033	0.016	0.057	**0.220**	0.050
Proteobacteria	*Alphaproteobacteria*		0.017	0.002	0.028	0.013	0.031	0.000	**0.164**	**0.192**
Proteobacteria	*Alphaproteobacteria*	*Micropepsis*	0.002	0.000	0.000	0.000	0.000	0.050	0.092	0.050
Bacteroidota	*Bacteroidia*	*Mucilaginibacter*	**0.111**	0.012	0.092	**0.112**	**0.155**	**0.149**	**1.834**	**1.003**
Proteobacteria	*Alphaproteobacteria*	*Uncultured Magnetospirillaceae*	0.000	0.000	0.000	0.000	0.000	0.036	0.036	0.036
Planctomycetota	*Planctomycetes*	*Uncultured Gemmataceae*	0.059	0.000	0.000	0.053	0.000	**0.135**	0.100	0.064
Proteobacteria	*Gammaproteobacteria*	*Burkholderia-Caballeronia-Paraburkholderia*	**0.808**	**0.130**	**0.576**	**1.818**	**1.039**	**2.112**	**13.346**	**25.092**
Planctomycetota	*Planctomycetes*	*Paludisphaera*	0.000	0.000	0.000	0.000	0.000	0.000	0.000	0.014
Campilobacterota	*Campylobacteria*	*Sulfuricurvum*	0.000	0.000	0.000	0.007	0.000	0.000	0.000	**0.420**
Actinobacteriota	*Actinobacteria*	*Pseudonocardia*	0.000	0.000	0.000	0.000	0.000	0.000	0.000	0.007
Planctomycetota	*Phycisphaerae*	*WD2101_soil_group (F)*	0.021	0.005	0.000	0.053	0.008	0.050	**0.135**	0.092
Proteobacteria	*Gammaproteobacteria*	*Aquicella*	0.000	0.000	0.000	0.000	0.000	0.007	0.071	0.007
Deinococcota	*Deinococci*	*Truepera*	0.000	0.000	0.000	0.000	0.000	**0.178**	0.000	0.000
Patescibacteria	*Saccharimonadia*		0.069	0.002	0.007	0.026	0.039	0.021	**0.185**	**0.121**

Proteobacteria	*Gammaproteobacteria*	*Massilia*	0.012	0.017	**0.142**	**0.217**	**0.535**	**0.377**	**1.187**	**0.270**
Actinobacteriota	*Thermoleophilia*	*Uncultured Gaiellales*	0.036	0.017	0.021	0.079	**0.178**	**0.107**	**0.576**	**0.363**
Proteobacteria	*Alphaproteobacteria*		0.073	0.019	0.092	0.059	0.031	**0.206**	**1.223**	**0.896**
Actinobacteriota	*Actinobacteria*	*Nakamurella*	0.000	0.002	0.000	0.000	0.000	0.000	0.007	0.036
Proteobacteria	*Alphaproteobacteria*	*Uncultured Acetobacteraceae*	0.024	0.002	0.014	0.039	0.000	0.057	**0.540**	**0.420**
Elusimicrobiota	*Elusimicrobia*	*Lineage_IV (F)*	0.017	0.009	0.092	0.000	0.016	0.085	**0.412**	**0.299**
Proteobacteria	*Gammaproteobacteria*	*Rhodanobacter*	**0.666**	**0.123**	**0.675**	**0.624**	**0.636**	**1.187**	**5.738**	**2.304**
Actinobacteriota	*Actinobacteria*	*Leifsonia*	0.005	0.000	0.000	0.000	0.000	0.000	0.014	0.014
Armatimonadota	*Fimbriimonadia*	*Fimbriimonadaceae (F)*	0.007	0.002	0.007	0.020	0.016	0.021	**0.107**	**0.128**
Proteobacteria	*Alphaproteobacteria*	*Uncultured Parvibaculaceae (F)*	0.000	0.000	0.000	0.000	0.000	0.000	0.007	0.000
Actinobacteriota	*Actinobacteria*	*Streptomyces*	**0.922**	**0.116**	**0.754**	**0.899**	**0.659**	**1.735**	**2.098**	**0.697**
Actinobacteriota	*Acidimicrobiia*	*IMCC26256 (F)*	0.005	0.000	0.000	0.000	0.023	0.000	0.014	0.064
Chloroflexi			0.000	0.000	0.000	0.007	0.000	0.007	0.000	0.000
Acidobacteriota	*Acidobacteriae*	*Granulicella*	0.000	0.002	0.000	0.013	0.031	0.000	**0.199**	**0.206**
Verrucomicrobiota	*Verrucomicrobiae*	*Lacunisphaera*	0.009	0.000	0.000	0.000	0.000	0.014	0.057	0.036
Proteobacteria	*Gammaproteobacteria*	*Arsenophonus*	0.000	0.000	0.000	**0.269**	0.000	0.036	0.000	0.000
Firmicutes	*Bacilli*	*Alicyclobacillus*	0.000	0.000	0.000	0.000	0.008	**0.185**	0.021	0.007
Proteobacteria	*Alphaproteobacteria*		0.017	0.005	0.000	**0.171**	0.062	**0.220**	**0.434**	**0.491**
Actinobacteriota	*Actinobacteria*	*Jatrophihabitans*	0.000	0.000	0.007	0.000	0.031	0.064	**0.128**	**0.142**
Deinococcota	*Deinococci*	*Deinococcus*	0.000	0.000	0.000	0.007	0.054	**0.149**	0.000	0.000
Proteobacteria	*Alphaproteobacteria*		0.000	0.000	0.000	0.000	0.000	0.000	0.064	0.078
Patescibacteria	*Saccharimonadia*	*LWQ8 (F)*	0.050	0.014	0.007	**0.427**	0.078	**0.277**	**1.387**	**0.690**
Chloroflexi	*Chloroflexia*	*Nitrolancea*	0.000	0.002	0.000	0.000	0.000	0.000	0.021	0.036
Actinobacteriota	*Actinobacteria*	*Brevibacterium*	0.000	0.000	0.000	0.000	0.000	0.000	0.000	0.007
Bacteroidota	*Bacteroidia*	*Arachidicoccus*	0.017	0.014	0.007	0.092	0.008	0.007	**0.242**	**0.192**
Verrucomicrobiota	*Lentisphaeria*	*Arctic95B-14 (F)*	0.000	0.000	0.000	0.000	0.000	0.000	0.014	0.000

Proteobacteria	*Alphaproteobacteria*		0.026	0.002	0.021	0.020	0.047	0.043	0.078	0.071
Proteobacteria	*Alphaproteobacteria*	*Uncultured Micropepsaceae*	**0.258**	0.043	**0.128**	**0.308**	**0.209**	**0.427**	**3.065**	**2.887**
Proteobacteria	*Gammaproteobacteria*	*Pseudomonas*	**0.389**	**14.778**	**1.557**	**3.977**	**3.529**	**9.549**	**0.647**	**0.391**
Actinobacteriota	*Actinobacteria*		0.000	0.000	0.000	0.007	**0.116**	0.014	**0.142**	0.078
Actinobacteriota	*Actinobacteria*	*Rothia*	0.007	0.005	0.092	0.020	**0.256**	**0.277**	0.000	0.007
Firmicutes	*Clostridia*	*Lachnotalea*	0.005	0.000	0.000	0.000	0.000	0.000	0.014	**0.128**
Proteobacteria	*Alphaproteobacteria*	*Methylovirgula*	0.009	0.000	0.007	0.000	0.000	0.050	**0.270**	**0.156**
Firmicutes	*Bacilli*	*Oxalophagus*	0.000	0.000	0.000	0.000	0.000	0.014	0.000	0.000
Proteobacteria	*Gammaproteobacteria*	*Thermomonas*	0.000	0.000	0.000	0.000	0.000	0.000	0.021	0.000
Firmicutes	*Bacilli*	*Streptococcus*	0.000	0.000	0.000	0.072	0.000	0.014	0.000	0.000
Patescibacteria	*Saccharimonadia*	*Saccharimonadales (O)*	0.026	0.007	0.000	0.026	**0.357**	0.000	**0.491**	**0.299**
Actinobacteriota	*Actinobacteria*	*Cellulomonas*	0.007	0.000	0.064	0.026	0.062	0.085	**0.263**	0.085
Proteobacteria	*Gammaproteobacteria*	*Acidibacter*	0.088	0.007	0.085	**0.151**	**0.217**	**0.171**	**1.756**	**1.578**
Gemmatimonadota	*Gemmatimonadetes*	*Uncultured Gemmatimonadaceae*	0.012	0.000	0.000	0.053	0.000	0.014	0.043	0.050
Bacteroidota	*Bacteroidia*	*Hymenobacter*	0.000	0.000	0.000	0.000	0.000	0.092	0.000	0.000
Proteobacteria	*Alphaproteobacteria*	*Novosphingobium*	0.026	0.000	0.000	0.020	**0.209**	0.050	0.078	**0.142**
Planctomycetota	*Planctomycetes*	*Aquisphaera*	0.009	0.000	0.000	0.000	0.000	0.000	0.050	0.071
Acidobacteriota	*Acidobacteriae*	*Subgroup_13 (F)*	0.005	0.002	0.000	0.007	0.016	0.014	0.043	0.021
Bacteroidota	*Bacteroidia*	*Haoranjiania*	0.000	0.000	0.000	0.000	0.000	0.000	0.000	0.007
Proteobacteria	*Gammaproteobacteria*	*Steroidobacter*	0.012	0.002	0.028	0.026	0.008	0.092	0.028	0.021
Chloroflexi	*Ktedonobacteria*	*Uncultured Ktedonobacteraceae*	0.062	0.009	0.043	0.046	0.054	**0.228**	**0.981**	**0.114**
Proteobacteria	*Gammaproteobacteria*	*Dyella*	0.000	0.002	0.000	0.020	0.000	0.007	**0.220**	**0.213**
Proteobacteria	*Gammaproteobacteria*		0.005	0.017	**3.057**	**2.855**	**2.187**	**1.273**	0.050	**0.228**
Acidobacteriota	*Acidobacteriae*	*Occallatibacter*	0.009	0.007	0.064	0.066	0.023	**0.128**	**0.498**	**0.484**

Bdellovibrionota	*Oligoflexia*	*0319-6G20 (F)*	0.009	0.000	0.000	0.007	0.000	0.007	0.014	0.014
Acidobacteriota	*Acidobacteriae*	*Acidobacterium*	0.002	0.000	0.000	0.000	0.000	0.000	0.007	0.050
Proteobacteria	*Gammaproteobacteria*	*KF-JG30-C25 (F)*	0.019	0.017	0.014	0.033	0.039	0.064	**0.448**	**0.391**
Proteobacteria	*Gammaproteobacteria*	*SC-I-84 (F)*	0.007	0.000	0.000	0.026	0.000	0.000	0.071	0.014
Chloroflexi	*Dehalococcoidia*	*S085 (F)*	0.002	0.005	0.000	0.007	0.000	0.007	0.007	0.000
Firmicutes	*Bacilli*	*Anaerobacillus*	0.002	0.000	0.014	0.007	0.000	0.014	0.000	0.000
Proteobacteria	*Alphaproteobacteria*	*Defluviicoccus*	0.000	0.000	0.000	0.000	0.000	0.000	0.007	0.000
Proteobacteria	*Alphaproteobacteria*	*Asticcacaulis*	**0.308**	0.038	**0.270**	**0.545**	**0.271**	**0.484**	**2.119**	**1.977**
Proteobacteria	*Alphaproteobacteria*	*Methylobacterium-Methylorubrum*	0.000	0.000	0.000	0.000	0.023	0.036	0.000	0.007
Proteobacteria	*Alphaproteobacteria*	*Acidocella*	0.000	0.000	0.000	0.000	0.008	0.000	0.000	**0.284**
Verrucomicrobiota	*Chlamydiae*	*cvE6 (F)*	0.002	0.000	0.000	0.000	0.000	0.000	0.028	0.000
Proteobacteria	*Alphaproteobacteria*	*Candidatus_Finniella*	0.000	0.002	0.000	0.000	0.008	0.000	0.000	0.000
Chloroflexi	*Ktedonobacteria*	*B10-SB3A (F)*	0.000	0.000	0.000	0.000	0.000	0.014	0.000	0.000
Proteobacteria	*Alphaproteobacteria*		0.005	0.005	0.014	0.000	0.039	0.064	**0.725**	**0.277**
Bacteroidota	*Bacteroidia*	*Puia*	0.009	0.002	0.000	0.007	0.000	0.000	0.092	0.014
Proteobacteria	*Gammaproteobacteria*		0.000	0.000	0.000	0.007	0.000	0.000	0.000	0.057
Proteobacteria	*Alphaproteobacteria*	*Hephaestia*	**0.135**	0.009	0.078	**0.144**	**0.109**	**0.128**	**0.995**	**0.853**
Proteobacteria	*Gammaproteobacteria*	*Aeromonas*	0.000	0.000	0.000	0.000	0.000	0.000	0.000	0.021
Firmicutes	*Bacilli*	*Paenibacillus*	**71.101**	**73.590**	**0.434**	**0.991**	**1.730**	**1.052**	0.014	0.050
Patescibacteria	*Saccharimonadia*	*WWH38 (F)*	0.043	0.005	0.014	**0.144**	0.062	**0.178**	**0.306**	**0.107**
Proteobacteria			0.000	0.000	0.000	0.000	0.000	0.000	0.043	0.021
Proteobacteria	*Alphaproteobacteria*	*Sphingomonas*	0.002	0.000	0.028	0.066	**0.178**	**0.149**	**0.213**	0.092
Gemmatimonadota	*Gemmatimonadetes*	*Gemmatimonas*	0.038	0.000	0.021	0.039	0.000	0.028	**0.213**	**0.178**
Actinobacteriota	*Thermoleophilia*	*67-14 (F)*	0.028	0.002	0.028	0.000	0.000	0.071	**0.256**	**0.121**

Proteobacteria	*Alphaproteobacteria*	*Roseiarcus*	0.000	0.005	0.014	0.013	0.000	0.000	**0.142**	0.050
Proteobacteria	*Alphaproteobacteria*	*Uncultured Caulobacteraceae*	0.002	0.000	0.000	0.000	0.000	0.000	0.000	0.036
Cyanobacteria	*Vampirivibrionia*	*Obscuribacteraceae (F)*	0.009	0.009	0.000	0.046	0.039	0.014	**0.149**	**0.178**
Firmicutes	*Bacilli*	*Pullulanibacillus*	0.026	0.000	0.000	0.000	0.000	0.000	0.000	0.000
Acidobacteriota	*Vicinamibacteria*		0.000	0.000	0.000	0.000	0.000	0.000	0.036	0.021
Acidobacteriota	*Acidobacteriae*	*Edaphobacter*	0.000	0.002	0.000	0.000	0.000	0.000	0.036	0.021
Firmicutes	*Bacilli*	*Tumebacillus*	0.000	0.000	0.000	0.026	0.000	0.050	0.000	0.000
Proteobacteria	*Gammaproteobacteria*		0.033	0.002	0.057	0.000	0.008	0.050	**0.164**	**0.220**
Proteobacteria	*Gammaproteobacteria*	*JG36-TzT-191 (F)*	0.017	0.000	0.000	0.033	0.047	0.057	**0.398**	**0.356**
Dependentiae	*Babeliae*	*UBA12409 (F)*	0.002	0.000	0.000	0.000	0.000	0.000	0.028	0.000
Proteobacteria	*Alphaproteobacteria*	*Rhodopila*	0.000	0.000	0.000	0.000	0.000	0.000	0.014	0.014
Bacteroidota	*Bacteroidia*	*Edaphobaculum*	0.002	0.000	0.036	0.033	0.000	0.028	**0.220**	0.050
Proteobacteria	*Gammaproteobacteria*	*Ellin6067*	0.009	0.000	0.000	0.000	0.016	0.000	0.007	0.000
Myxococcota	*Polyangia*	*BIrii41 (F)*	0.000	0.007	0.000	0.007	0.000	0.007	0.000	0.000
Actinobacteriota	*Actinobacteria*	*Propionicimonas*	0.000	0.000	0.000	0.000	0.000	0.000	0.021	0.000
Firmicutes	*Bacilli*	*Bacillus*	**3.086**	**1.178**	0.021	**0.308**	**0.318**	**0.832**	0.021	0.021
Proteobacteria	*Alphaproteobacteria*	*Uncultured Micavibrionaceae*	0.007	0.000	0.000	0.000	0.000	0.021	0.007	0.000
Planctomycetota	*Phycisphaerae*	*CPla-3_termite_group*	0.090	0.002	0.007	0.039	0.023	0.028	0.085	0.036
Actinobacteriota	*Actinobacteria*		0.000	0.000	0.000	0.000	0.000	0.000	0.000	0.014
Actinobacteriota	*Thermoleophilia*	*Conexibacter*	0.021	0.000	0.000	0.013	0.000	0.014	**0.114**	0.050
Proteobacteria	*Alphaproteobacteria*	*Hyphomicrobium*	0.083	0.017	0.085	0.092	0.078	**0.185**	**1.038**	**1.223**
Proteobacteria	*Alphaproteobacteria*	*Sphingobium*	0.000	0.000	0.000	0.000	0.000	0.000	0.021	0.014
Acidobacteriota	*Acidobacteriae*	*Subgroup_2 (F)*	0.014	0.002	0.014	0.000	0.016	0.028	0.050	0.043
Bdellovibrionota	*Bdellovibrionia*	*Bdellovibrio*	0.014	0.005	0.000	0.000	0.016	0.014	**0.149**	0.078
Verrucomicrobiota	*Verrucomicrobiae*	*Ellin516*	0.002	0.000	**0.114**	0.000	0.000	0.036	0.085	0.043
Bacteroidota	*Bacteroidia*	*Cytophaga*	0.000	0.000	0.000	0.000	0.000	0.000	0.014	0.007

Acidobacteriota	*Acidobacteriae*		0.000	0.000	0.000	0.000	0.000	0.000	0.014	0.014
Proteobacteria	*Gammaproteobacteria*	*Coxiella*	0.000	0.000	0.000	0.000	0.000	0.000	0.007	0.000
Proteobacteria	*Alphaproteobacteria*	*Uncultured Zavarziniales*	0.002	0.000	0.000	0.000	0.000	0.000	0.000	0.000
Firmicutes	*Clostridia*	*Clostridium_sensu_stricto_8*	0.000	0.000	0.000	0.013	0.000	0.000	0.000	0.014
Bacteroidota	*Bacteroidia*	*Uncultured*	0.064	0.014	0.036	**0.131**	0.023	0.064	**0.341**	**0.292**
Proteobacteria	*Alphaproteobacteria*	*Telmatospirillum*	0.000	0.000	0.000	0.000	0.000	0.000	0.014	0.014
Proteobacteria	*Gammaproteobacteria*	*TRA3-20 (F)*	0.014	0.005	0.000	0.000	0.000	0.078	**0.356**	**0.327**
Actinobacteriota	*Acidimicrobiia*	*Uncultured Acidimicrobiia*	0.002	0.000	0.000	0.007	0.000	0.014	0.007	0.036
Proteobacteria	*Alphaproteobacteria*	*Xanthobacteraceae (F)*	0.000	0.000	0.000	0.000	0.000	0.021	0.000	0.000
Bacteroidota	*Bacteroidia*	*Pedobacter*	0.000	0.000	0.000	0.000	0.000	0.000	0.043	0.000
Bacteroidota	*Bacteroidia*	*Niastella*	0.000	0.000	0.000	0.000	0.000	0.000	0.036	0.000
Spirochaetota	*Spirochaetia*	*Spirochaeta*	0.009	0.000	0.000	0.000	0.000	0.057	0.014	0.043
Dependentiae	*Babeliae*		0.002	0.000	0.000	0.000	0.000	0.000	0.028	0.007
Firmicutes	*Clostridia*	*Fonticella*	0.000	0.000	0.000	0.000	0.000	0.021	0.021	0.064
Proteobacteria	*Alphaproteobacteria*		0.000	0.000	0.000	0.000	0.000	0.000	0.000	0.028
Actinobacteriota	*Actinobacteria*	*Mycobacterium*	0.005	0.002	0.050	0.000	0.054	0.085	**0.114**	**0.142**
Firmicutes	*Bacilli*		0.021	0.000	0.007	0.007	0.054	0.014	0.085	**0.220**
Acidobacteriota	*Acidobacteriae*		0.012	0.000	0.000	0.000	0.047	0.007	**0.185**	**0.199**
Firmicutes	*Bacilli*	*Lactobacillus*	0.000	0.000	0.000	0.020	0.000	0.014	0.000	0.014
Proteobacteria	*Gammaproteobacteria*	*Delftia*	0.000	0.000	0.028	0.013	0.000	0.007	0.000	0.000
Proteobacteria	*Gammaproteobacteria*		**0.130**	0.052	**68.757**	**45.760**	**42.809**	**24.801**	0.007	**0.547**
Cyanobacteria	*Vampirivibrionia*	*Vampirovibrionaceae (F)*	0.005	0.002	0.000	0.000	0.000	0.000	0.000	0.000
Bacteroidota	*Bacteroidia*	*37-13 (F)*	0.002	0.000	0.043	0.000	0.000	0.000	0.007	0.000
Proteobacteria	*Alphaproteobacteria*	*NRL2 (F)*	0.000	0.000	0.000	0.000	0.000	0.000	0.000	0.021
Bacteroidota	*Bacteroidia*	*Sporocytophaga*	0.012	0.000	0.000	0.007	0.023	0.014	0.021	0.021

Proteobacteria	*Gammaproteobacteria*	*Alkanibacter*	0.000	0.000	0.000	0.000	0.000	0.000	0.007	0.014
Chloroflexi	*KD4-96*	*KD4-96 (F)*	0.007	0.000	0.000	0.000	0.000	0.000	0.057	0.028
Proteobacteria	*Gammaproteobacteria*	*Chujaibacter*	0.059	0.002	0.050	**0.125**	0.070	**0.192**	**1.301**	**1.045**
Proteobacteria	*Alphaproteobacteria*	*Acidicaldus*	0.000	0.000	0.000	0.007	0.000	0.000	0.071	**0.142**
Proteobacteria	*Alphaproteobacteria*	*Devosia*	**0.142**	0.017	**0.121**	**0.197**	**0.124**	**0.363**	**0.846**	**0.633**
Acidobacteriota	*Acidobacteriae*	*Candidatus_Solibacter*	0.002	0.000	0.000	0.000	0.000	0.000	0.036	0.014
Firmicutes	*Sulfobacillia*	*Uncultured Sulfobacillaceae*	0.000	0.000	0.000	0.007	0.008	0.000	0.000	0.028
Proteobacteria	*Alphaproteobacteria*	*Pseudolabrys*	**0.137**	0.019	0.078	**0.269**	**0.132**	**0.284**	**1.223**	**0.789**
Bacteroidota	*Bacteroidia*	*Wandonia*	0.017	0.005	0.000	0.000	0.000	0.100	0.028	0.028
Verrucomicrobiota	*Verrucomicrobiae*	*Opitutus*	0.078	0.005	0.057	0.072	0.016	**0.164**	**0.263**	**0.277**
FCPU426	*FCPU426*	*FCPU426 (F)*	0.066	0.014	0.100	0.072	**0.155**	**0.114**	**0.292**	**0.306**
Firmicutes	*Negativicutes*	*Uncultured Veillonellales-Selenomonadales*	0.000	0.000	0.000	0.000	0.000	0.000	0.000	0.085
Patescibacteria	*Saccharimonadia*	*TM7a*	0.000	0.002	0.000	0.013	0.000	0.014	0.028	0.000
Actinobacteriota	*Actinobacteria*	*Sinomonas*	0.000	0.000	0.000	0.000	0.000	0.028	0.000	0.000
Verrucomicrobiota	*Omnitrophia*	*Omnitrophales (O)*	0.000	0.000	0.000	0.000	0.000	0.000	0.000	0.007
Acidobacteriota	*Vicinamibacteria*	*Uncultured Vicinamibacterales*	0.033	0.000	0.021	0.020	0.000	0.057	0.100	0.043
Proteobacteria	*Alphaproteobacteria*	*Phenylobacterium*	0.024	0.009	0.021	**0.125**	0.031	0.036	**0.156**	**0.171**
Actinobacteriota	*Actinobacteria*	*Gryllotalpicola*	0.000	0.002	0.000	0.000	0.000	0.000	0.036	0.064
Firmicutes	*Clostridia*		0.000	0.000	0.000	0.013	0.008	0.014	**0.306**	**0.391**
Gemmatimonadota	*Gemmatimonadetes*		0.002	0.000	0.000	0.033	0.000	0.007	0.021	0.000
Proteobacteria	*Gammaproteobacteria*	*EPR3968-O8a-Bc78 (F)*	0.000	0.000	0.000	0.000	0.000	0.000	0.007	0.007
Actinobacteriota	*Acidimicrobiia*		0.000	0.000	0.000	0.020	0.008	0.036	0.021	0.021
Proteobacteria	*Gammaproteobacteria*	*Legionella*	0.007	0.000	0.000	0.046	0.000	0.000	0.057	0.007
Chloroflexi	*TK10*	*TK10 (F)*	0.000	0.000	0.000	0.000	0.000	0.000	0.014	0.000
Proteobacteria	*Alphaproteobacteria*		0.000	0.000	0.000	0.000	0.000	0.000	0.000	0.007

Firmicutes	*Bacilli*		0.000	0.005	0.000	0.000	0.000	0.000	0.000	0.000
Firmicutes	*Clostridia*		0.000	0.000	0.000	0.000	0.047	0.000	0.036	0.036
Proteobacteria	*Alphaproteobacteria*	*Caulobacter*	0.000	0.000	0.000	0.000	0.000	0.000	**0.370**	0.000
Proteobacteria	*Alphaproteobacteria*	*Uncultured Methyloligellaceae*	0.000	0.000	0.000	0.000	0.000	0.000	0.028	0.000
Proteobacteria	*Alphaproteobacteria*	*Methyloligella*	0.000	0.000	0.000	0.007	0.000	0.007	0.050	**0.107**
Bacteroidota	*Bacteroidia*	*Bacteroides*	0.000	0.000	0.000	0.000	0.000	0.000	0.000	**0.263**
Bdellovibrionota	*Bdellovibrionia*	*OM27_clade*	0.000	0.000	0.000	0.000	0.000	0.000	0.000	0.007
Proteobacteria	*Gammaproteobacteria*	*Azotobacter*	0.000	0.000	0.000	0.000	0.000	0.000	0.000	**0.107**
Proteobacteria	*Alphaproteobacteria*	*Dongia*	**0.119**	0.007	0.071	0.085	0.000	**0.213**	**0.910**	**0.675**
Proteobacteria	*Gammaproteobacteria*	*Candidatus_Portiera*	0.000	0.002	0.007	**2.179**	0.062	**0.476**	0.000	0.000
Verrucomicrobiota	*Chlamydiae*	*Uncultured Simkaniaceae*	0.002	0.000	0.000	0.000	0.008	0.000	0.064	0.007
Actinobacteriota	*Actinobacteria*	*Corynebacterium*	0.000	0.000	0.000	0.013	0.023	0.050	0.000	0.000
Firmicutes	*Desulfitobacteriia*	*Desulfosporosinus*	0.002	0.000	0.000	0.000	0.016	0.014	0.021	**0.739**
Myxococcota	*Polyangia*		0.000	0.000	0.000	0.000	0.000	0.000	0.014	0.000
Bacteroidota	*Bacteroidia*	*Dysgonomonas*	0.000	0.000	0.000	0.000	0.000	0.000	0.014	0.000
Proteobacteria	*Gammaproteobacteria*	*Uncultured Diplorickettsiaceae*	0.000	0.000	0.000	0.000	0.023	0.021	0.028	0.036
Actinobacteriota	*Actinobacteria*	*Flexivirga*	0.005	0.000	0.000	0.013	0.000	0.014	0.021	0.021
WPS-2	*WPS-2*	*WPS-2 (F)*	0.000	0.000	0.007	0.000	0.000	0.000	0.064	0.021
Proteobacteria	*Gammaproteobacteria*	*Noviherbaspirillum*	0.002	0.000	0.000	0.000	0.000	0.000	0.036	**0.107**
Proteobacteria	*Gammaproteobacteria*	*Thiomonas*	0.002	0.000	0.000	0.007	0.000	0.007	0.100	**1.301**
Actinobacteriota	*Actinobacteria*		0.000	0.000	0.000	0.033	0.000	0.007	**0.142**	**0.135**
Proteobacteria	*Gammaproteobacteria*	*Uliginosibacterium*	**3.190**	**0.543**	**2.830**	**3.925**	**2.909**	**5.624**	**10.438**	**6.662**
Bacteroidota	*Bacteroidia*		0.009	0.002	0.092	0.072	0.016	0.050	**0.135**	**0.228**
Proteobacteria	*Alphaproteobacteria*		0.021	0.000	0.014	0.000	0.000	0.078	0.057	0.064

Proteobacteria	*Alphaproteobacteria*	*Uncultured Kiloniellaceae*	0.000	0.000	0.000	0.033	0.000	0.000	0.000	0.007
Proteobacteria	*Gammaproteobacteria*	*Buchnera*	0.000	0.000	0.043	**0.532**	**2.699**	**0.256**	0.000	0.000
Planctomycetota	*Phycisphaerae*	*I-8*	0.000	0.000	0.000	0.000	0.000	0.000	0.000	0.007
Proteobacteria	*Alphaproteobacteria*	*Afipia*	0.000	0.000	0.000	0.072	0.023	0.000	**0.178**	0.071
Proteobacteria	*Alphaproteobacteria*	*D05-2 (F)*	0.021	0.000	0.000	0.000	0.000	0.007	**0.156**	0.092
Myxococcota	*Myxococcia*	*Anaeromyxobacter*	0.012	0.007	0.000	0.007	0.016	0.014	0.007	0.000
Firmicutes	*Clostridia*	*Clostridium_sensu_stricto_9*	0.000	0.000	0.000	0.000	0.008	0.021	0.028	**0.164**
Proteobacteria	*Gammaproteobacteria*	*Acinetobacter*	0.009	0.000	0.021	0.020	0.023	0.092	0.000	0.000
Desulfobacterota	*Desulfuromonadia*	*Citrifermentans*	0.000	0.005	0.000	0.000	0.000	0.007	0.100	**0.540**
Firmicutes	*Clostridia*	*Mobilitalea*	0.000	0.000	0.000	0.013	0.016	0.007	0.000	0.000
Patescibacteria	*Saccharimonadia*	*Saccharimonadaceae (F)*	0.000	0.000	0.000	0.000	0.000	0.000	0.000	0.007
Firmicutes	*Bacilli*	*Cohnella*	**9.118**	**3.197**	0.014	0.000	0.008	0.000	0.000	0.000
Abditibacteriota	*Abditibacteria*	*Abditibacterium*	0.000	0.000	0.000	0.000	0.000	0.000	0.000	0.014
Actinobacteriota	*Actinobacteria*	*Curtobacterium*	0.005	0.000	0.021	0.000	0.070	**0.149**	0.007	0.000
Firmicutes	*Clostridia*	*Clostridium_sensu_stricto_1*	0.047	0.002	0.014	0.053	0.031	0.092	**0.939**	**4.060**
Patescibacteria	*Parcubacteria*	*Parcubacteria (C)*	0.000	0.000	0.000	0.000	0.000	0.000	0.007	0.000
Firmicutes	*Negativicutes*	*Sporomusa*	0.000	0.000	0.000	0.000	0.000	0.000	0.071	0.007
Myxococcota	*Polyangia*	*Haliangium*	0.000	0.000	0.000	0.000	0.008	0.007	0.014	0.000
Actinobacteriota	*Actinobacteria*	*Arthrobacter*	0.007	0.000	0.000	0.000	0.000	0.028	0.000	0.000
Firmicutes	*Negativicutes*	*Uncultured Sporomusaceae*	0.000	0.000	0.000	0.000	0.000	0.000	0.000	0.007
Proteobacteria	*Alphaproteobacteria*	*Altererythrobacter*	0.000	0.000	0.000	0.000	0.000	0.000	0.007	0.007
Spirochaetota	*Spirochaetia*	*Spirochaeta_2*	0.062	0.000	0.043	0.026	0.008	0.057	**0.156**	**0.121**
Patescibacteria	*Parcubacteria*	*Candidatus_Nomurabacteria (F)*	0.007	0.000	0.000	0.000	0.000	0.000	0.000	0.000
Proteobacteria	*Gammaproteobacteria*	*Mizugakiibacter*	0.000	0.000	0.000	0.000	0.000	0.000	0.014	0.007
Chloroflexi	*JG30-KF-CM66*	*JG30-KF-CM66 (F)*	0.000	0.000	0.028	0.000	0.000	0.000	0.000	0.007
Actinobacteriota	*Actinobacteria*	*Actinospica*	0.005	0.000	0.028	0.013	0.054	0.036	**0.199**	**0.178**

Actinobacteriota	*Actinobacteria*	*Humibacter*	0.000	0.000	0.000	0.013	0.000	0.000	0.057	0.092
Planctomycetota	*Planctomycetes*		0.000	0.000	0.000	0.000	0.016	**0.185**	0.007	0.014
Proteobacteria	*Alphaproteobacteria*	*Inquilinus*	0.000	0.000	0.000	0.000	0.000	0.000	0.064	0.021
Proteobacteria	*Gammaproteobacteria*	*Rhodanobacteraceae (F)*	0.000	0.000	0.000	0.000	0.000	0.000	0.007	0.000
Firmicutes	*Bacilli*	*Geobacillus*	0.000	0.000	0.000	0.000	0.000	0.000	0.000	0.007
Proteobacteria	*Gammaproteobacteria*	*MM2*	0.000	0.000	0.000	0.000	0.000	0.000	**0.476**	0.000
Firmicutes	*Clostridia*	*Herbinix*	0.000	0.000	0.000	0.000	0.000	0.000	0.000	0.007
Firmicutes			0.000	0.000	0.000	0.000	0.000	0.064	0.014	0.000
Firmicutes	*Clostridia*	*Lachnoclostridium*	0.000	0.000	0.000	0.000	0.000	0.021	0.085	**0.164**
Proteobacteria	*Alphaproteobacteria*	*Rhodopseudomonas*	0.000	0.000	0.000	0.000	0.000	0.007	0.078	0.021
Bacteroidota	*Bacteroidia*	*Spirosoma*	0.000	0.000	0.000	0.072	0.000	0.000	0.000	0.000
Bacteroidota	*Bacteroidia*	*Uncultured Microscillaceae*	0.040	0.002	0.007	0.066	0.054	0.021	**0.306**	**0.206**
Myxococcota	*Myxococcia*		0.000	0.000	0.000	0.000	0.023	0.000	0.007	0.000
Planctomycetota	*Planctomycetes*	*Singulisphaera*	0.014	0.000	0.000	0.000	0.000	0.000	0.014	0.021
Bacteroidota	*Bacteroidia*		0.000	0.000	0.000	0.000	0.000	0.000	0.007	0.000
Firmicutes	*Clostridia*	*Anaerovorax*	0.000	0.000	0.000	0.007	0.000	0.000	0.014	**0.263**
Proteobacteria	*Alphaproteobacteria*	*Rhizomicrobium*	0.017	0.000	0.000	0.020	0.000	0.021	0.071	0.000
Proteobacteria	*Gammaproteobacteria*	*[Polyangium]_brachysporum_group*	0.000	0.000	0.000	0.000	0.000	0.000	0.000	0.021
Planctomycetota	*Planctomycetes*	*Pir4_lineage*	0.000	0.000	0.014	0.000	0.000	0.000	0.000	0.000
Bacteroidota	*Bacteroidia*	*NS9_marine_group*	0.000	0.000	0.000	0.000	0.000	0.000	0.036	0.000
Proteobacteria	*Gammaproteobacteria*	*Dokdonella*	0.017	0.000	0.014	0.000	0.016	0.021	0.036	0.028
Myxococcota	*Polyangia*	*Pajaroellobacter*	0.000	0.000	0.014	0.000	0.016	0.000	0.000	0.000
Myxococcota	*Myxococcia*	*P3OB-42*	0.000	0.000	0.000	0.000	0.000	0.000	0.014	0.007
Proteobacteria	*Alphaproteobacteria*	*Uncultured Rickettsiaceae*	0.000	0.000	0.000	0.000	0.000	0.014	0.007	0.028
Verrucomicrobiota	*Verrucomicrobiae*	*Candidatus_Udaeobacter*	0.000	0.000	0.014	0.007	0.000	0.014	0.021	0.000

Proteobacteria	*Alphaproteobacteria*	*Allorhizobium-Neorhizobium-Pararhizobium-Rhizobium*	0.002	0.000	0.014	0.046	0.016	0.071	**3.342**	**0.505**
Bacteroidota	*Bacteroidia*	*Uncultured Chitinophagales*	0.000	0.000	0.000	0.013	0.047	0.000	0.007	0.000
Proteobacteria	*Alphaproteobacteria*	*Wolbachia*	0.000	0.000	0.028	**2.363**	0.000	**0.135**	0.000	0.000
Proteobacteria	*Gammaproteobacteria*		0.000	0.000	**0.448**	0.085	0.000	**0.192**	0.000	0.000
Firmicutes	*Clostridia*	*Caproiciproducens*	0.000	0.000	0.000	0.000	0.000	0.000	0.000	0.007
Bacteroidota	*Bacteroidia*	*Fluviicola*	0.000	0.000	0.000	0.000	0.000	0.000	0.021	0.000
Proteobacteria	*Gammaproteobacteria*		0.069	0.017	0.014	**0.269**	**0.155**	**0.256**	**1.387**	**1.877**
Planctomycetota	*Planctomycetes*		0.000	0.000	0.000	0.033	0.000	0.000	0.000	0.007
Bacteroidota	*Bacteroidia*		0.019	0.000	0.000	0.000	0.016	0.000	0.064	0.028
Verrucomicrobiota	*Chlamydiae*		0.000	0.000	0.000	0.007	0.000	0.000	0.000	0.000
Desulfobacterota	*Desulfuromonadia*		0.000	0.000	0.000	0.033	0.000	0.014	**0.135**	**0.327**
Verrucomicrobiota	*Verrucomicrobiae*	*Uncultured Opitutaceae*	0.019	0.007	0.007	0.046	0.016	**0.156**	**0.220**	**0.192**
Proteobacteria	*Gammaproteobacteria*	*Uncultured Neisseriaceae*	0.000	0.000	0.000	0.000	0.008	0.007	0.000	0.000
Actinobacteriota	*Actinobacteria*		0.002	0.000	0.000	0.000	0.000	0.000	0.043	0.000
Firmicutes	*Clostridia*	*Clostridium_sensu_stricto_12*	0.000	0.000	0.000	0.000	0.000	0.000	0.000	0.057
Planctomycetota	*Planctomycetes*	*Uncultured Isosphaeraceae*	0.000	0.000	0.000	0.000	0.000	0.000	0.000	0.014
Bacteroidota	*Bacteroidia*	*Taibaiella*	0.014	0.000	0.000	0.046	0.000	0.021	0.100	0.085
Proteobacteria	*Alphaproteobacteria*	*Mesorhizobium*	0.024	0.002	0.007	0.007	0.000	0.014	**0.121**	0.036
Cyanobacteria	*Vampirivibrionia*	*Vampirovibrionales (O)*	0.000	0.000	0.007	0.000	0.000	0.000	0.000	0.000
Proteobacteria	*Gammaproteobacteria*	*CCD24 (F)*	0.000	0.000	0.000	0.000	0.000	0.000	0.007	0.000
Bacteroidota	*Bacteroidia*	*Siphonobacter*	0.000	0.000	0.000	0.007	0.000	0.043	0.000	0.000
Proteobacteria	*Alphaproteobacteria*	*Uncultured Magnetospiraceae*	0.021	0.000	0.028	0.079	0.000	0.000	0.036	0.028
Proteobacteria	*Gammaproteobacteria*	*CHAB-XI-27 (F)*	0.002	0.000	0.000	0.000	0.000	0.000	0.007	0.000
Proteobacteria	*Alphaproteobacteria*	*SWB02*	0.002	0.002	0.000	0.020	0.000	0.000	0.071	0.000

Actinobacteriota	*Thermoleophilia*		0.000	0.000	0.000	0.000	0.000	0.021	0.000	0.000
Proteobacteria	*Gammaproteobacteria*	*MND1*	0.021	0.009	0.021	0.000	0.000	0.007	**0.135**	0.028
Proteobacteria	*Alphaproteobacteria*	*Uncultured Xanthobacteraceae*	0.000	0.002	0.000	0.000	0.000	0.000	0.028	0.043
Proteobacteria	*Alphaproteobacteria*	*Bauldia*	0.000	0.000	0.000	0.000	0.039	0.000	0.028	0.057
Chloroflexi	*Ktedonobacteria*	*FCPS473*	0.007	0.005	0.000	0.059	0.000	0.000	0.092	0.000
Proteobacteria	*Gammaproteobacteria*	*Castellaniella*	0.002	0.007	0.000	0.000	0.000	0.021	**0.576**	**0.171**
Bacteroidota	*Bacteroidia*	*CWT_CU03-E12 (F)*	0.005	0.000	0.007	0.000	0.000	0.007	0.078	0.028
Actinobacteriota	*Thermoleophilia*		0.000	0.000	0.000	0.000	0.000	0.021	0.000	0.000
Actinobacteriota	*Actinobacteria*	*Diaminobutyricimonas*	0.000	0.000	0.000	0.007	0.000	0.000	0.000	0.007
Proteobacteria	*Alphaproteobacteria*	*Uncultured Rhodospirillaceae*	0.007	0.002	0.000	0.000	0.000	0.000	0.000	0.007
Actinobacteriota	*Actinobacteria*	*Micromonospora*	0.000	0.000	0.000	0.000	0.000	0.000	0.014	0.014
Proteobacteria	*Gammaproteobacteria*	*Uncultured Moraxellaceae*	0.000	0.000	0.000	0.000	0.000	0.000	0.007	0.000
Firmicutes	*Thermaerobacteria*	*Thermaerobacter*	0.000	0.000	0.000	0.000	0.000	0.007	0.000	0.000
Chloroflexi	*Chloroflexia*	*JG30-KF-CM45 (F)*	0.000	0.000	0.000	0.000	0.000	0.000	0.000	0.021
Proteobacteria	*Gammaproteobacteria*	*Nitrosospira*	0.000	0.000	0.000	0.000	0.000	0.000	0.007	0.000
Proteobacteria	*Gammaproteobacteria*	*Enhydrobacter*	0.000	0.000	0.021	0.053	0.000	0.000	0.000	0.000
Patescibacteria	*Parcubacteria*	*Candidatus_Jorgensenbacteria (F)*	0.002	0.000	0.000	0.020	0.000	0.000	0.007	0.021
Proteobacteria	*Alphaproteobacteria*	*Acidiphilium*	0.000	0.000	0.000	0.000	0.000	0.014	0.000	0.036
Firmicutes	*Clostridia*	*Anaerocolumna*	0.000	0.000	0.000	0.000	0.000	0.000	0.036	**0.164**
Actinobacteriota	*Thermoleophilia*	*JCM_18997*	0.000	0.002	0.000	0.013	0.000	0.000	0.007	0.000
Proteobacteria	*Gammaproteobacteria*	*Alcanivorax*	0.000	0.000	0.000	0.033	0.000	0.000	0.000	0.007
Cyanobacteria	*Sericytochromatia*	*Sericytochromatia*	0.000	0.000	0.021	0.000	0.000	0.000	0.021	0.007
Firmicutes	*Clostridia*	*Clostridium_sensu_stricto_10*	0.000	0.000	0.000	0.000	0.000	0.000	0.007	0.000

Proteobacteria	*Gammaproteobacteria*	*Candidatus_Ovatusbacter*	0.000	0.002	0.000	0.000	0.000	0.000	0.007	0.036
Acidobacteriota	*Acidobacteriae*	*Candidatus_Koribacter*	0.000	0.000	0.000	0.000	0.000	0.000	0.057	0.028
Actinobacteriota	*Actinobacteria*		0.000	0.000	0.000	0.000	0.000	0.000	0.021	0.000
Campilobacterota	*Campylobacteria*	*Sulfurospirillum*	0.000	0.000	0.000	0.000	0.000	0.000	0.000	0.036
Proteobacteria	*Gammaproteobacteria*		0.000	0.000	0.000	0.007	0.008	0.000	0.000	0.000
Proteobacteria	*Alphaproteobacteria*	*Uncultured Defluviicoccales*	0.000	0.000	0.000	0.000	0.000	0.000	0.007	0.000
Actinobacteriota	*Actinobacteria*	*Actinoplanes*	**0.168**	0.036	**0.178**	**0.243**	**0.132**	**0.299**	0.028	0.036
Cyanobacteria	*Vampirivibrionia*	*Gastranaerophilales (O)*	0.000	0.000	0.000	0.000	0.000	0.000	0.000	0.007
Chloroflexi	*Ktedonobacteria*	*C0119 (F)*	0.000	0.002	0.000	0.000	0.039	0.000	0.036	0.014
Proteobacteria	*Alphaproteobacteria*	*Bradyrhizobium*	0.014	0.000	0.085	0.007	0.070	**0.156**	**0.391**	**0.220**
Bacteroidota	*Bacteroidia*	*env.OPS_17 (F)*	0.017	0.000	0.000	0.000	0.008	0.036	0.071	0.014
Proteobacteria	*Gammaproteobacteria*	*Luteibacter*	0.000	0.000	0.000	0.013	0.016	0.007	0.007	0.021
Proteobacteria	*Alphaproteobacteria*		0.000	0.000	0.000	0.000	0.016	0.000	0.050	0.043
Proteobacteria	*Gammaproteobacteria*		0.000	0.000	0.000	0.000	0.000	0.000	0.000	0.007
Firmicutes	*Clostridia*		0.000	0.000	0.000	0.000	0.000	0.000	0.000	**0.107**
Bacteroidota	*Bacteroidia*	*KD3-93 (F)*	0.000	0.000	0.000	0.000	0.000	0.000	0.021	0.000
Actinobacteriota	*Actinobacteria*		0.000	0.000	0.000	0.000	0.000	0.000	0.021	0.021
Actinobacteriota	*Thermoleophilia*		0.000	0.000	0.000	0.000	0.000	0.000	0.007	0.000
Verrucomicrobiota	*Verrucomicrobiae*		0.000	0.000	0.000	0.007	0.000	0.000	0.007	0.000
Actinobacteriota	*Acidimicrobiia*	*Uncultured Ilumatobacteraceae*	0.000	0.000	0.000	0.000	0.000	0.014	0.021	0.000
Actinobacteriota	*Actinobacteria*	*Uncultured Sporichthyaceae*	0.000	0.000	0.000	0.000	0.000	0.000	0.021	0.000
Proteobacteria	*Alphaproteobacteria*	*Tistrella*	0.000	0.000	0.000	0.000	0.000	0.000	0.000	0.043
Proteobacteria	*Gammaproteobacteria*	*Thauera*	**0.116**	0.014	0.085	0.066	0.062	**0.185**	**1.123**	**0.185**
Firmicutes	*Desulfotomaculia*	*Desulfofarcimen*	0.000	0.000	0.000	0.000	0.000	0.014	0.000	0.000
Proteobacteria	*Alphaproteobacteria*	*Skermanella*	0.000	0.000	0.000	0.000	0.000	0.000	0.007	0.000

Actinobacteriota	*Actinobacteria*	*Microbacterium*	0.000	0.002	0.000	0.000	**0.240**	0.007	0.000	0.000
Actinobacteriota	*Actinobacteria*	*Frankia*	0.002	0.000	0.000	0.000	0.000	0.000	0.021	0.000
Patescibacteria	*Saccharimonadia*	*TM7*	0.000	0.000	0.000	0.000	0.000	0.000	0.014	0.000
Proteobacteria	*Alphaproteobacteria*	*Pseudaminobacter*	0.000	0.000	0.000	0.000	0.000	0.000	0.014	0.036
Proteobacteria	*Alphaproteobacteria*	*Azospirillum*	0.007	0.005	0.007	0.026	0.008	0.078	**0.448**	0.007
Planctomycetota	*Planctomycetes*	*Uncultured Planctomycetales*	0.007	0.000	0.014	0.007	0.000	0.000	0.085	0.021
Proteobacteria	*Alphaproteobacteria*	*Rhodoplanes*	0.002	0.000	0.000	0.000	0.000	0.000	0.000	0.007
Actinobacteriota	*Actinobacteria*	*Cutibacterium*	0.014	0.002	**0.107**	0.092	**0.349**	**0.462**	0.000	0.021
Proteobacteria	*Alphaproteobacteria*		0.002	0.000	0.000	0.000	0.000	0.000	0.014	0.000
Proteobacteria	*Alphaproteobacteria*	*Parvibaculum*	0.007	0.002	0.000	0.053	0.000	0.007	**0.114**	**0.164**
Proteobacteria	*Alphaproteobacteria*	*Nitrospirillum*	0.000	0.000	0.036	**0.190**	0.000	0.028	0.000	**0.156**
Actinobacteriota	*Actinobacteria*		0.000	0.000	0.000	0.000	0.000	0.050	0.007	0.000
Proteobacteria	*Alphaproteobacteria*	*Sandaracinobacter*	0.002	0.002	0.000	0.013	0.000	0.028	0.071	0.057
Firmicutes	*Bacilli*	*Staphylococcus*	0.000	0.002	0.064	**0.112**	0.093	**0.228**	0.000	0.036
Proteobacteria	*Alphaproteobacteria*	*Uncultured*	0.000	0.005	0.000	0.000	0.000	0.000	0.000	0.000
Proteobacteria	*Alphaproteobacteria*		0.017	0.005	0.000	0.033	0.000	0.050	**0.235**	**0.256**
Proteobacteria	*Gammaproteobacteria*	*WD260 (F)*	0.007	0.000	0.000	0.000	0.000	0.000	0.000	0.000
Firmicutes	*Clostridia*	*Oxobacter*	0.000	0.000	0.000	0.000	0.000	0.000	0.000	0.007
Proteobacteria	*Alphaproteobacteria*	*AB1 (F)*	0.000	0.000	0.000	0.000	0.000	0.000	0.007	0.000
Chloroflexi	*Ktedonobacteria*		0.000	0.000	0.043	0.000	0.023	0.036	**0.149**	0.028
Cyanobacteria	*Cyanobacteriia*	*Uncultured Chroococcidiopsaceae*	0.000	0.000	0.000	0.000	0.016	**0.263**	0.000	0.000
Firmicutes	*Bacilli*	*Ammoniphilus*	0.031	0.000	0.000	0.007	0.039	0.014	0.021	0.028
Proteobacteria	*Gammaproteobacteria*	*Halomonas*	0.007	0.005	0.007	0.007	0.000	0.057	0.007	0.007
Proteobacteria	*Gammaproteobacteria*	*Luteimonas*	0.002	0.000	0.000	0.000	0.008	0.000	0.007	0.000
Proteobacteria	*Gammaproteobacteria*	*Uncultured Gammaproteobacteria*	0.000	0.000	0.000	0.000	0.000	0.000	0.007	0.000
Bacteroidota	*Bacteroidia*	*Chryseobacterium*	0.000	0.000	0.007	0.000	0.000	0.000	0.100	0.000

Firmicutes	*Thermoanaerobacteria*	*Thermoanaerobacterium*	0.000	0.000	0.000	0.000	0.000	0.000	0.007	0.000
Actinobacteriota	*Actinobacteria*	*Aestuariimicrobium*	0.007	0.000	0.000	0.000	0.000	0.021	0.000	0.000
Proteobacteria	*Gammaproteobacteria*	*Methylophilus*	0.000	0.000	0.000	0.007	0.000	0.000	**0.626**	**0.533**
Proteobacteria	*Alphaproteobacteria*	*Uncultured Paracaedibacteraceae*	0.000	0.000	0.000	0.000	0.000	0.000	0.000	0.007
RCP2-54	*RCP2-54*	*RCP2-54 (F)*	0.002	0.000	0.000	0.000	0.000	0.000	0.000	0.000
Verrucomicrobiota	*Verrucomicrobiae*	*Prosthecobacter*	0.000	0.007	0.043	0.000	0.000	0.000	0.050	0.100
Bacteroidota	*Bacteroidia*	*Flavisolibacter*	0.000	0.000	0.000	0.000	0.000	0.000	0.036	0.000
Patescibacteria	*Saccharimonadia*		0.000	0.000	0.050	0.007	0.000	0.014	0.100	0.043
Proteobacteria	*Alphaproteobacteria*	*Chthonobacter*	0.000	0.000	0.000	0.000	0.000	0.000	0.007	0.000
Proteobacteria	*Alphaproteobacteria*	*Acidisoma*	0.000	0.000	0.000	0.000	0.000	0.000	0.014	0.050
Chloroflexi	*OLB14*	*OLB14 (F)*	0.002	0.000	0.000	0.000	0.000	0.007	0.028	0.007
Actinobacteriota	*Actinobacteria*	*Geodermatophilus*	0.000	0.000	0.000	0.000	0.000	0.014	0.014	0.014
Proteobacteria	*Gammaproteobacteria*	*Curvibacter*	0.000	0.000	0.000	0.000	0.000	0.043	0.000	0.000
Verrucomicrobiota	*Verrucomicrobiae*	*Pedosphaeraceae (F)*	0.000	0.000	0.000	0.013	0.000	0.000	0.000	0.000
Proteobacteria	*Gammaproteobacteria*	*Herbaspirillum*	0.000	0.000	0.000	0.000	0.000	0.000	0.000	0.014
Bacteroidota	*Ignavibacteria*	*UA-50 (F)*	0.009	0.000	0.000	0.013	0.000	0.014	0.000	0.000
Firmicutes	*Clostridia*	*Clostridia_UCG-014*	0.000	0.000	0.000	0.000	0.000	0.000	0.000	0.028
Actinobacteriota	*Actinobacteria*	*Dermacoccus*	0.000	0.000	0.000	0.007	0.000	0.000	0.000	0.000
Proteobacteria	*Gammaproteobacteria*	*Cupriavidus*	0.000	0.000	0.014	0.007	0.031	0.007	0.000	0.000
Proteobacteria	*Gammaproteobacteria*	*Cellvibrio*	0.000	0.000	0.000	0.000	0.000	0.000	0.000	0.014
Firmicutes	*Bacilli*	*Lysinibacillus*	0.000	0.000	0.000	0.000	0.000	0.021	0.000	0.000
Actinobacteriota	*Actinobacteria*	*Lawsonella*	0.000	0.000	0.000	0.000	0.016	0.028	0.000	0.000
Patescibacteria	*Saccharimonadia*	*Candidatus_Saccharimonas*	0.000	0.000	0.000	0.000	0.000	0.000	0.000	**0.114**
Firmicutes	*Negativicutes*	*Pelosinus*	0.000	0.000	0.000	0.000	0.000	0.028	0.028	**0.114**

Proteobacteria	*Gammaproteobacteria*	*Procabacter*	0.000	0.000	0.000	0.000	0.016	0.000	0.000	0.000
Firmicutes	*Negativicutes*	*Anaerosinus*	0.002	0.002	0.000	0.000	0.000	0.007	**1.664**	0.071
Firmicutes	*Bacilli*	*Erysipelothrix*	0.000	0.000	0.000	0.000	0.000	0.007	0.000	0.000
Actinobacteriota	*Actinobacteria*	*Micrococcus*	0.002	0.000	0.000	0.013	0.000	0.028	0.000	0.000
Actinobacteriota	*Actinobacteria*		0.000	0.000	0.000	0.000	0.000	0.000	0.014	0.000
Firmicutes	*Bacilli*	*Planifilum*	0.000	0.000	0.000	0.020	0.008	0.000	0.000	0.000
Actinobacteriota	*Acidimicrobiia*	*Uncultured Microtrichales*	0.000	0.000	0.000	0.000	0.000	0.000	0.000	0.007
Proteobacteria	*Gammaproteobacteria*	*Halothiobacillus*	0.000	0.000	0.000	0.000	0.000	0.000	0.000	0.050
Bacteroidota	*Bacteroidia*	*Paludibacter*	0.000	0.000	0.000	0.000	0.000	0.014	0.000	0.000
Desulfobacterota	*Uncultured Desulfobacterota*	*Uncultured Desulfobacterota*	0.005	0.000	0.007	0.000	0.000	0.000	0.000	0.000
Proteobacteria	*Alphaproteobacteria*	*Reyranella*	0.000	0.000	0.043	0.000	0.000	0.000	0.000	0.007
Proteobacteria	*Alphaproteobacteria*	*Pseudovibrio*	0.000	0.005	0.007	0.000	0.000	0.000	0.021	0.028
Actinobacteriota	*Actinobacteria*		0.000	0.000	0.000	0.000	0.008	0.000	0.014	0.043
Actinobacteriota	*Actinobacteria*	*Rhodococcus*	0.000	0.000	0.000	0.000	0.000	0.007	0.000	0.000
Acidobacteriota	*Acidobacteriae*	*Terracidiphilus*	0.000	0.000	0.000	0.000	0.000	0.021	0.021	0.000
Firmicutes	*Clostridia*	*Gracilibacter*	0.000	0.000	0.000	0.000	0.000	0.000	0.014	0.000
Actinobacteriota	*Actinobacteria*	*Kocuria*	0.000	0.000	0.000	0.013	0.023	0.000	0.007	0.000
Proteobacteria	*Gammaproteobacteria*	*Escherichia-Shigella*	0.012	0.000	0.007	0.013	0.000	**0.199**	0.000	0.000
Firmicutes	*Clostridia*	*Clostridium_sensu_stricto_13*	0.000	0.002	0.000	0.000	0.000	0.000	0.000	0.007
Proteobacteria	*Alphaproteobacteria*	*Rhodovastum*	0.005	0.000	0.000	0.000	0.000	0.014	0.014	0.043
Proteobacteria	*Gammaproteobacteria*		0.000	0.000	0.000	0.000	0.000	0.000	0.000	0.007
Spirochaetota	*Spirochaetia*		0.000	0.000	0.000	0.000	0.000	0.000	0.000	0.007
Proteobacteria	*Gammaproteobacteria*	*Ottowia*	0.000	0.000	0.000	0.000	0.000	0.000	0.036	0.007

Table S5. *P*-values of the comparison between *G. clandestina* seed samples belonging to G1 and G2 (T and C) seed. Significant differences were determined using the Kruskal Wallis pairwise test using Alpha diversity (Observed features) and are highlighted in bold.

**Group 1**	**Group 2**	***p*-Value**
G1 seed	G2 seed (T)	0.220
G1 seed	G2 seed (C)	0.133
G2 seed (T)	G2 seed (C)	**0.000**

Table S6. *P*-values of the comparison between *G. clandestina* seed samples belonging to G1 and G2 (T and C) seed. Significant differences were determined using the pairwise-ANOSIM test using Beta diversity (Jaccard distance) metrics and are highlighted in bold.

**Group 1**	**Group 2**	***R***	***p*-Value**
G1 seed	G2 seed (T)	0.671559	**0.001**
G1 seed	G2 seed (C)	0.672733	**0.001**
G2 seed (T)	G2 seed (C)	0.33925	**0.001**

Table S7. *P*-values of the comparison between G1 plant organs of *G. clandestina* belonging to root, shoot and leaf and G2 seed grown in two soil treatments (T and C). Significant differences were determined using the Kruskal Wallis pairwise test for Alpha diversity (Observed features) and are highlighted in bold.

**Group 1**	**Group 2**	***p*-Value**
Leaf (T)	Leaf (C)	0.192
Leaf (T)	Root (T)	**0.000**
Leaf (T)	Root (C)	**0.000**
Leaf (T)	Shoot (T)	0.171
Leaf (T)	Shoot (C)	**0.009**
Leaf (T)	G2 seed (T)	0.071
Leaf (T)	G2 seed (C)	0.655
Leaf (C)	Root (T)	**0.000**
Leaf (C)	Root (C)	**0.000**
Leaf (C)	Shoot (T)	0.954
Leaf (C)	Shoot (C)	0.053
Leaf (C)	G2 seed (T)	0.171
Leaf (C)	G2 seed (C)	**0.021**
Root (T)	Root (C)	0.954
Root (T)	Shoot (T)	**0.000**
Root (T)	Shoot (C)	**0.000**
Root (T)	G2 seed (T)	**0.000**
Root (T)	G2 seed (C)	**0.000**
Root (C)	Shoot (T)	**0.000**
Root (C)	Shoot (C)	**0.000**
Root (C)	G2 seed (T)	**0.000**
Root (C)	G2 seed (C)	**0.000**
Shoot (T)	Shoot (C)	**0.021**
Shoot (T)	G2 seed (T)	0.326

Shoot (T)	G2 seed (C)	0.154
Shoot (C)	G2 seed (T)	**0.010**
Shoot (C)	G2 seed (C)	**0.000**
G2 seed (T)	G2 seed (C)	**0.000**

Table S8. *P*-values of the comparison between G1 plant organs of *G. clandestina* belonging to root, shoot and leaf and G2 seed grown in two soil treatments (T and C). Significant differences were determined using pairwise-ANOSIM test using Beta diversity (Jaccard distance) metrics and are highlighted in bold.

**Group 1**	**Group 2**	***R***	***p*-Value**
Leaf (T)	Leaf (C)	0.098	0.073
Leaf (T)	Root (T)	0.859	**0.001**
Leaf (T)	Root (C)	0.864	**0.001**
Leaf (T)	Shoot (T)	0.059	0.159
Leaf (T)	Shoot (C)	0.243	**0.018**
Leaf (T)	G2 seed (T)	0.763	**0.001**
Leaf (T)	G2 seed (C)	0.691	**0.001**
Leaf (C)	Root (T)	0.713	**0.001**
Leaf (C)	Root (C)	0.705	**0.001**
Leaf (C)	Shoot (T)	0.069	0.131
Leaf (C)	Shoot (C)	**0.027**	0.238
Leaf (C)	G2 seed (T)	0.673	**0.001**
Leaf (C)	G2 seed (C)	0.727	**0.001**
Root (T)	Root (C)	0.219	**0.008**

Root (T)	Shoot (T)	0.840	**0.001**
Root (T)	Shoot (C)	0.695	**0.001**
Root (T)	G2 seed (T)	0.990	**0.001**
Root (T)	G2 seed (C)	0.997	**0.001**
Root (C)	Shoot (T)	0.846	**0.001**
Root (C)	Shoot (C)	0.718	**0.001**
Root (C)	G2 seed (T)	0.988	**0.001**
Root (C)	G2 seed (C)	0.995	**0.001**
Shoot (T)	Shoot (C)	0.176	0.057
Shoot (T)	G2 seed (T)	0.786	**0.001**
Shoot (T)	G2 seed (C)	0.705	**0.001**
Shoot (C)	G2 seed (T)	0.742	**0.001**
Shoot (C)	G2 seed (C)	0.779	**0.001**
G2 seed (T)	G2 seed (C)	0.367	**0.001**

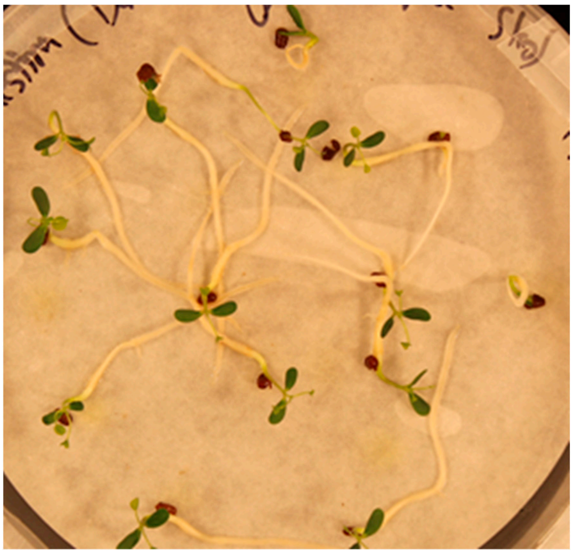

Figure S1. *G. clandestina* seedlings at the unfolded cotyledon growth stage.

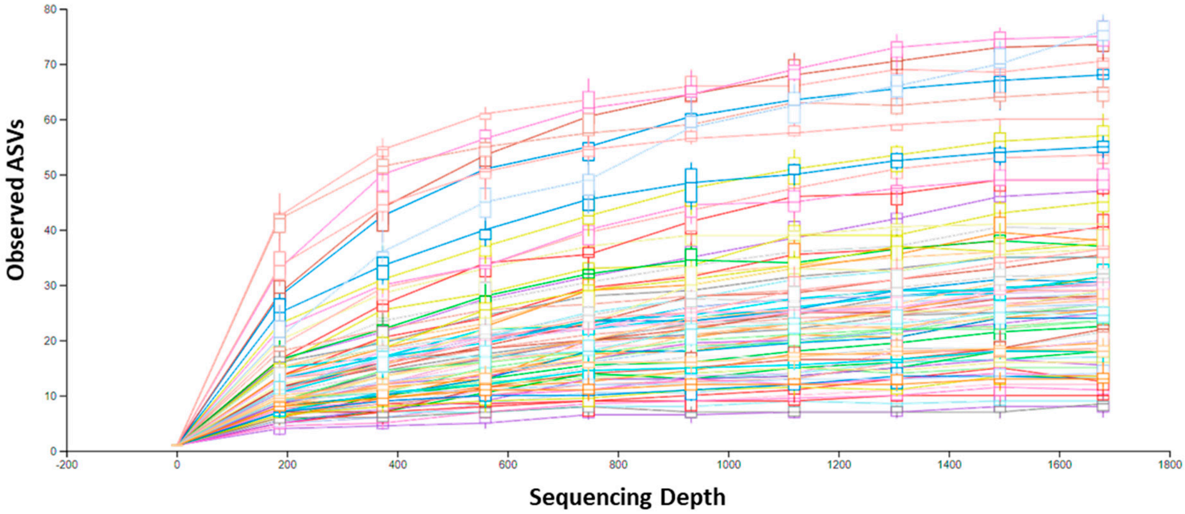

Figure S2. Rarefaction curves showing the number of observed ASVs at a sampling depth of 1680 sequences when data was grouped by soil treatment for microbiome profiling of *G. clandestina* seed. Each coloured line represents one sample.

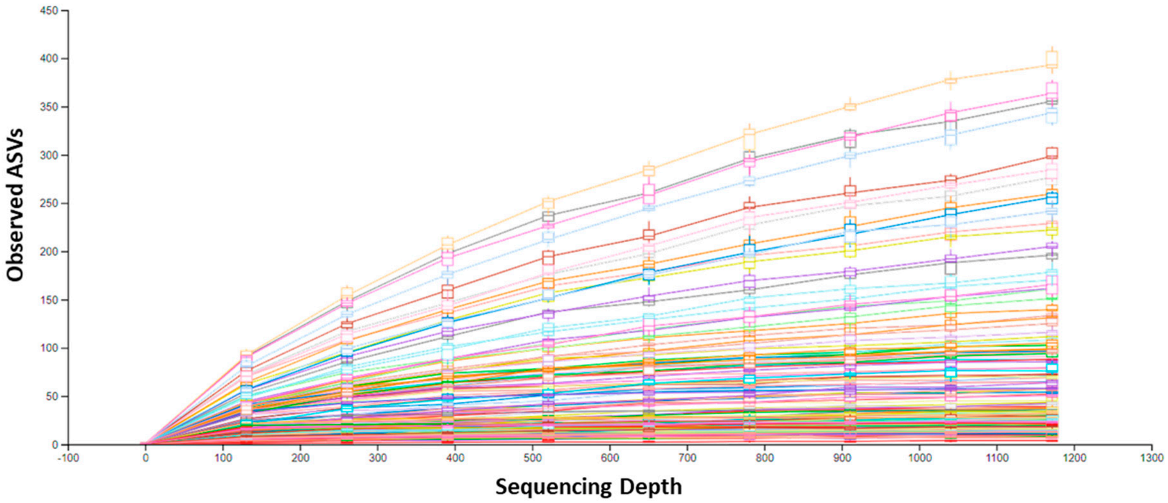

Figure S3. Rarefaction curves showing the number of observed ASVs at a sampling depth of 1172 sequences when data was grouped by soil treatment for microbiome profiling of *G. clandestina* plant organs. Each coloured line represents one sample.
